# Frequency patterns of semantic change: corpus-based evidence of a near-critical dynamics in language change

**DOI:** 10.1098/rsos.170830

**Published:** 2017-11-08

**Authors:** Q. Feltgen, B. Fagard, J.-P. Nadal

**Affiliations:** 1 Laboratoire de Physique Statistique, École Normale Supérieure, PSL Research University; Université Paris Diderot, Sorbonne Paris-Cité; Sorbonne Universités, UPMC—Univ. Paris 06; CNRS, Paris, France; 2 Laboratoire Langues, Textes, Traitements informatique, Cognition (Lattice, CNRS, ENS & Université Paris 3, PSL & USPC), École normale supérieure, Paris, France; 3 École des Hautes Études en Sciences Sociales, PSL Research University, CNRS, Centre d’Analyse et de Mathématique Sociales, Paris, France

**Keywords:** language change, grammaticalization, language modelling, S-curve, corpus based

## Abstract

It is generally believed that when a linguistic item acquires a new meaning, its overall frequency of use rises with time with an S-shaped growth curve. Yet, this claim has only been supported by a limited number of case studies. In this paper, we provide the first corpus-based large-scale confirmation of the S-curve in language change. Moreover, we uncover another generic pattern, a latency phase preceding the S-growth, during which the frequency remains close to constant. We propose a usage-based model which predicts both phases, the latency and the S-growth. The driving mechanism is a random walk in the space of frequency of use. The underlying deterministic dynamics highlights the role of a control parameter which tunes the system at the vicinity of a saddle-node bifurcation. In the neighbourhood of the critical point, the latency phase corresponds to the diffusion time over the critical region, and the S-growth to the fast convergence that follows. The durations of the two phases are computed as specific first-passage times, leading to distributions that fit well the ones extracted from our dataset. We argue that our results are not specific to the studied corpus, but apply to semantic change in general.

## 1. Introduction

Language can be approached through three different, complementary perspectives. Ultimately, it exists in the mind of language users, so that it is a cognitive entity, rooted in a neuropsychological basis. But language exists only because people interact with each other: It corresponds to a convention among a community of speakers, and answers to their communicative needs. Thirdly, language can be seen as something in itself: An autonomous, emergent entity, obeying its own inner logic. If it was not for this third Dasein of language, it would be less obvious to speak of language change as such.

The social and cognitive nature of language informs and constrains this inner consistency. Zipf’s Law, for instance, may be seen as resulting from a trade-off between the ease of producing the utterance, and the ease of processing it [1]. It relies thus both on the cognitive grounding of the language, and on its communicative nature. Those two external facets of language, cognitive and sociological, are similarly expected to channel the regularities of linguistic change. Modelling attempts (see [2] for an overview) have explored both how sociolinguistic factors can shape the process of this change [3,4] and how this change arises through language learning by new generations of users [5,6]. Some models also consider mutations of language itself, without providing further details on the social or cognitive mechanisms of change [7]. In this paper, we adopt the view that language change is initiated by language use, which is the repeated call to one’s linguistic resources in order to express oneself or to make sense of the linguistic productions of others. This approach is in line with exemplar models [8] and related works, such as the Utterance Selection Model [9] or the model proposed by Victorri [10], which describes an out-of-equilibrium shaping of semantic structure through repeated events of communication.

Leaving aside sociolinguistic factors, we focus on a cognitive approach of linguistic change, more precisely of semantic expansion. Semantic expansion occurs when a new meaning is gained by a word or a construction (we will henceforth refer more vaguely to a linguistic ‘form’, so as to remain as general as possible). For instance, *way*, in the construction *way too*, has come to serve as an intensifier (e.g. ‘The only other newspaper in the history of Neopia is the Ugga Ugg Times, which, of course, is way too prehistoric to read.’ [11]). The fact that polysemy is pervasive in any language [12] suggests that semantic expansion is a common process of language change and happens constantly throughout the history of a language. Grammaticalization [13]—a process by which forms acquire a (more) grammatical status, like the example of *way too* above—and other interesting phenomena of language change [14,15] fall within the scope of semantic expansion.

Semantic change is known to be associated with an increase in frequency of use of the form whose meaning expands. This increase is expected indeed: As the form comes to carry more meanings, it is used in a broader number of contexts, hence more often. This implies that any instance of semantic change should have its empirical counterpart in the frequency rise of the use of the form. This rise is furthermore believed to follow an S-curve. The main reference on this phenomenon remains undisputedly the work of Kroch [16], which unfortunately grounds his claim on a handful of examples only. It has nonetheless become an established fact in the literature of language change [17]. The origin of this pattern largely remained undiscussed, until recently: Blythe & Croft [18], in addition to an up-to-date aggregate survey of attested S-curve patterns in the literature (totalizing about 40 cases of language change), proposed a modelling account of the S-curve. However, they show that, in their framework, the novelty can rise only if it is deemed better than the old variant, a claim which clearly does not hold in all instances of language change. Their attempt also suffers, as most modelling works on the S-curve, from what is known as the Threshold Problem, the fact that a novelty will fail to take over an entire community of speakers, because of the isolated status of an exceptional deviation [19], unless a significant fraction of spontaneous adopters supports it initially.

On the other hand, the S-curve is not a universal pattern of frequency change in language. From a recent survey of the frequency evolution of 14 words relating to climate science [20], it appears that the S-curve could not account for most of the frequency changes, and that a more general Bass curve would be appropriate instead. Along the same line, Ghanbarnejad *et al.* [21] investigated 30 instances of language change: 10 regarding the regularization of tense in English verbs (e.g. cleave, clove, cloven > cleave, cleaved, cleaved), 12 relating to the transliteration of Russian names in English (e.g. Stroganoff > Stroganov) and eight to spelling changes in German words (ss>ß>ss) following two different ortographic reforms (in 1901 and 1996). They showed that the S-curve is not universal and that, in some cases, the trajectory of change rather obeys an exponential. This would be due to the preponderance of an external driving impetus over the other mechanisms of change, among which social imitation. The non-universality of the S-curve contrasts with the survey in [18], and is probably due to the specific nature of the investigated changes (which, for the spelling ones, relates mostly to academic conventions and affects very little the language system). This hypothesis would tend to be confirmed by the observation that, for the regularization of tense marking, an S-curve is observed most of the time (7 out of 10). It must also be stressed that none of these changes are semantic changes.

In this paper, we provide a broad corpus-based investigation of the frequency patterns associated with about 400 semantic expansions (about 10-fold the aggregate survey of Blythe & Croft [18]). It turns out that the S-curve pattern is corroborated, but must be completed by a preceding latency part, in which the frequency of the form does not significantly increase, even if the new meaning is already present in the language. This statistical survey also allows to obtain statistical distributions for the relevant quantities describing the S-curve pattern (the rate, width and length of the preceding latency part).

Apart from this data foraging, we provide a usage-based model of the process of semantic expansion, implementing basic cognitive hypotheses regarding language use. By means of our model, we relate the microprocess of language use at the individual scale, to the observed macro-phenomenon of a recurring frequency pattern occurring in semantic expansion. The merit of this model is to provide a unified theoretical picture of both the latency and the S-curve, which are understood in relation with Cognitive Linguistics notions such as inference and semantic organization. It also predicts that the statistical distributions for the latency time and for the growth time should be of the same family as the inverse Gaussian distribution, a claim which is in line with our data survey.

## 2. Quantifying change from corpus data

We worked on the French textual database *Frantext* [22], to our knowledge the only textual database allowing for a reliable study covering several centuries (see Material and methods and electronic supplementary material, SIII). We studied changes in frequency of use for 408 forms which have undergone one or several semantic expansions, on a time range going from 1321 up to the present day. We chose forms so as to focus on semantic expansions leading to a functional meaning—such as discursive, prepositional or procedural meanings. Semantic expansions whose outcome remains in the lexical realm (as the one undergone by *sentence*, whose meaning evolved from ‘verdict, judgment’ to ‘meaningful string of words’) have been left out. Functional meanings indeed present several advantages: They are often accompanied by a change of syntagmatic context, allowing to track the semantic expansion more accurately (e.g. *way* in *way too* + adj.); they are also less sensitive to sociocultural and historical influences; finally, they are less dependent on the specific content of a text, be it literary or academic.

**Figure 1.**
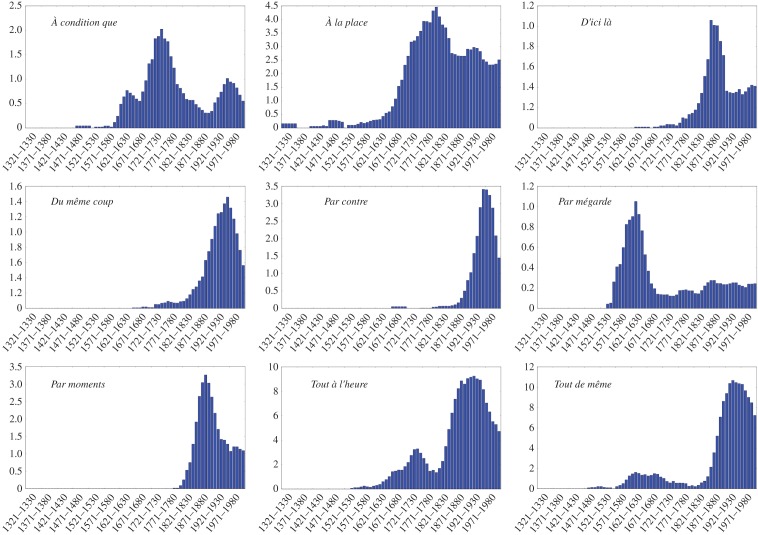
Frequency evolution on the whole time range (1321–2020) of nine different forms. Each blue bar shows the frequency associated with a decade. Frequency has been multiplied by a factor of 10^5^ for an easier reading.

**Figure 2.**
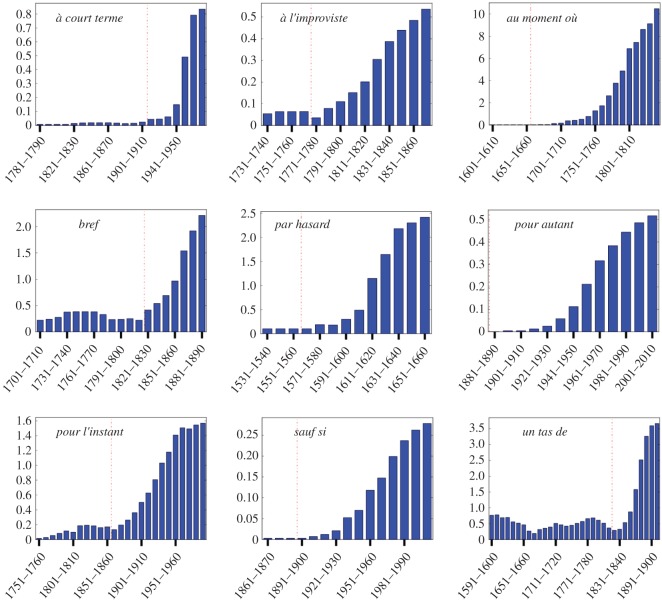
Extracted pattern of frequency rise for nine selected forms. The latency period and the S-growth are separated by a red vertical line.

**Figure 3.**
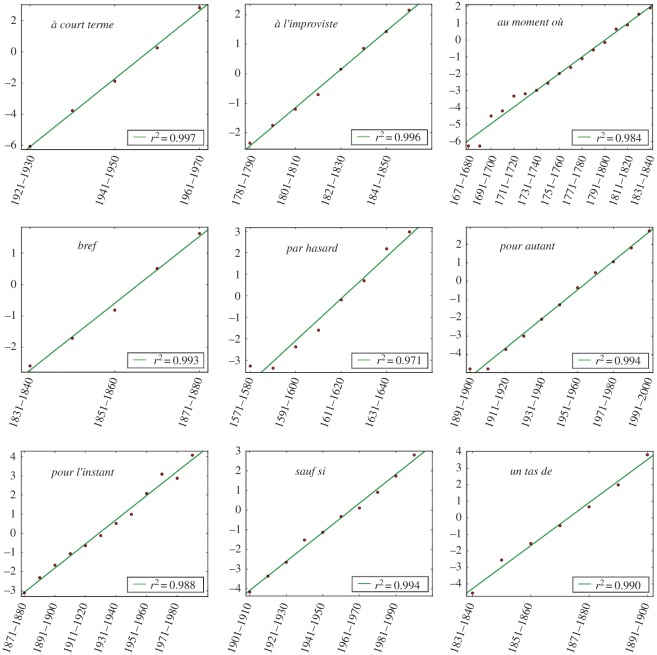
Logit transforms of the S-growth part of the preceding curves. Red dots correspond to data points and the green line to the linear fit of this set of points. The *r*^2^ coefficient of the linear fit is also displayed.

The profiles of frequency of use extracted from the database are illustrated on figure 1 for nine forms. We find that 295 cases (which makes up more than 70% of the total) display at least one sigmoidal increase of frequency in the course of their evolution, with a *p*-value significance of 0.05 compared to a random growth. We provide a small selection of the observed frequency patterns (figure 2), whose associated logit transforms (figure 3) follows a linear behaviour, indicative of the sigmoidal nature of the growth (see Material and methods). We thus find a robust statistical validation of the sigmoidal pattern, confirming the general claim made in the literature.

Furthermore, we find two major phenomena besides this sigmoidal pattern. The first one is that, in most cases, the final plateau towards which the frequency is expected to stabilize after its sigmoidal rise is not to be found: The frequency immediately starts to decrease after having reached a maximum (figure 1). However, such a decrease process is not symmetrical with the increase, in contrast with other cases of fashion-driven evolution in language, e.g. first names distribution [23]. Though this decrease may be, in a few handfuls of cases, imputable to the disappearance of a form (e.g. *après ce*, replaced in Modern French by *après quoi*), in most cases it is more likely to be the sign of a narrowing of its uses (equivalent, then, to a semantic depletion).

**Figure 4.**
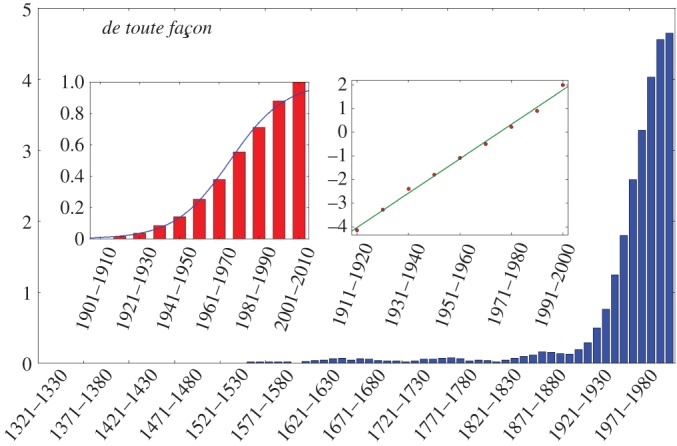
Overall evolution of the frequency of use of *de toute façon* (main panel), with focus on the S-shape increase (left inner panel), whose logit transformation follows a linear fit (right inner panel) with an *r*^2^ of 0.996. Preceding the S-growth, one observes a long period of very low frequency (up to 35 decades).

The second feature is that the fast growth is most often (in 69% of cases) preceded by a long latency up to several centuries, during which the new form is used, but with a comparatively low and rather stable frequency (figure 2). How the latency time is extracted from data is explained in Material and methods. One should note that the latency times may be underestimated: If the average frequency is very low during the latency part, the word may not show up at all in the corpus, especially in decades for which the available texts are sparse. The pattern of frequency increase is thus better conceived of as a latency followed by a growth, as exemplified by *de toute façon* (figure 4)—best translated by *anyway* in English, because the present meanings of these two terms are very close, and remarkably, despite quite different origins, the two have followed parallel paths of change.

To our knowledge, this latency feature has not been documented before, even though a number of specific cases of sporadic use of the novelty before the fast growth has been noticed. For instance, it has been remarked in the case of *just because* that the fast increase is only one stage in the evolution [24]. Other examples have been mentioned [25], but it was described there as the slow start of the sigmoid. On the other hand, the absence of a stable plateau has been observed and theorized as a ‘reversible change’ [26] or a ‘change reversal’ [27], and was seen as an occasional deviation from the usual S-curve, not as a pervasive phenomenal feature of the evolution. We rather interpret it as an effect of the constant interplay of forms in language, resulting in ever-changing boundaries for most of their respective semantic dominions.

In the following, we propose a model describing both the latency and the S-growth periods. The study of this decrease of frequency following the S-growth is left for future work.

## 3. Model

### 3.1. A cognitive scenario

To account for the specific frequency pattern evidenced by our data analysis, we propose a scenario focusing on cognitive aspects of language use, leaving all sociolinguistic effects backgrounded by making use of a representative agent, mean-field type, approach. We limit ourselves to the case of a competition between two linguistic variants, given that most cases of semantic expansion can be understood as such, even if the two competing variants cannot always be explicitly identified. Indeed, the variants need not be individual forms, and can be schematic constructions, paradigms of forms or abstract patterns. Furthermore, the competition is more likely to be local, and to involve a specific and limited region of the semantic territory. If the invaded form occupies a large semantic dominion, then losing a competition on its border will only affect its meaning marginally, so that the competition can fail to be perceptible from the point of view of the established form.

The idealized picture is therefore as such: Initially, in some concept or context of use *C*_1_, one of the two variants, henceforth noted as *Y* , is systematically chosen, so that it conventionally expresses this concept. The question we address is thus how a new variant, say *X*, can be used in this context and eventually evict the old variant *Y* ?

The main hypothesis we propose is that the new variant almost never is a brand new merging of phonemes whose meaning would pop out of nowhere. As Haspelmath highlights [28], a new variant is almost always a periphrastic construction, i.e. actual parts of language, put together in a new, meaningful way. Furthermore, such a construction, though it may be exapted to a new use, may have shown up from time to time in the time course of the language history, in an entirely compositional way; this is the case for *par ailleurs*, which incidentally appears as early as the fourteenth century in our corpus, but arises as a construction in its own right during the first part of the nineteenth century only. In other words, the use of a linguistic form *X* in a context *C*_1_ may be entirely new, but the form *X* was most probably already there in another context of use *C*_0_, or equivalently, with another meaning.

We make use of the well-grounded idea [29] that there exist links between concepts due to the intrinsic polysemy of language: There are no isolated meanings, as each concept is interwoven with many others, in a complicated tapestry. These links between concepts are asymmetrical, and they can express both universal mappings between concepts [30,31] and cultural ones (e.g. entrenched metaphors [32]). As the conceptual texture of language is a complex network of living relations rather than a collection of isolated and self-sufficient monads, semantic change is expected to happen as the natural course of language evolution and to occur repetitively throughout its history, so that at any point of time, there are always several parts of language which are undergoing changes. The simplest layout accounting for this network structure in a competitive situation consists then in two sites, such that one is influencing the other through a cognitive connexion of some sort.

### 3.2. Model formalism

We now provide details on the modelling of a competition between two variants *X* and *Y* for a given context of use, or concept, *C*_1_, also considering the effect exerted by the related context or concept *C*_0_ on this evolution.

— Each concept *C*_*i*_,*i*=0,1, is represented by a set of exemplars of the different linguistic forms. We note that $N_{\mu }^{i}(t)$ is the number at time *t* of encoded exemplars (or occurrences) of form *μ*∈{*X*,*Y* }, in context *C*_*i*_, in the memory of the representative agent.
— The memory capacity of an individual being finite, the population of exemplars attached to each concept *C*_*i*_ has a finite size *M*_*i*_. For simplicity we assume that all memory sizes are equal (*M*_0_=*M*_1_=*M*). As we consider only two forms *X* and *Y* , for each *i* the relation $N_{X}^{i}(t)+N_{Y}^{i}(t)=M$ always holds: We can focus on one of the two forms, here *X*, and drop out the form subscript, granted that all quantities refer to *X*.
— The absolute frequency $x_{t}^{i}$ of form *X* at time *t* in context *C*_*i*_—the fraction of ‘balls’ of type *X* in the bag attached to *C*_*i*_—is thus given by the ratio *N*^*i*^(*t*)/*M*. In the initial situation, *X* and *Y* are assumed to be established conventions for the expression of *C*_0_ and *C*_1_, respectively, so that we start with *N*^0^(*t*=0)=*M* and *N*^1^(*t*=0)=0.
— Finally, *C*_0_ exerts an influence on context *C*_1_, but this influence is assumed to be unilateral. Consequently, the content of *C*_0_ will not change in the course of the evolution and we can focus on *C*_1_. An absence of explicit indication of context is thus to be understood as referring to *C*_1_.

The dynamics of the system runs as follows. At each time *t*, one of the two linguistic forms is chosen to express concept *C*_1_. The form *X* is uttered with some probability *P*(*t*), to be specified below, and *Y* with probability 1−*P*(*t*). To keep constant the memory size of the population of occurrences in *C*_1_, a past occurrence is randomly chosen (with a uniform distribution) and the new occurrence takes its place. This dynamics is then repeated a large number of times. Note that this model focuses on a speaker perspective (for alternative variants, see electronic supplementary material, SIIA).

**Figure 5.**
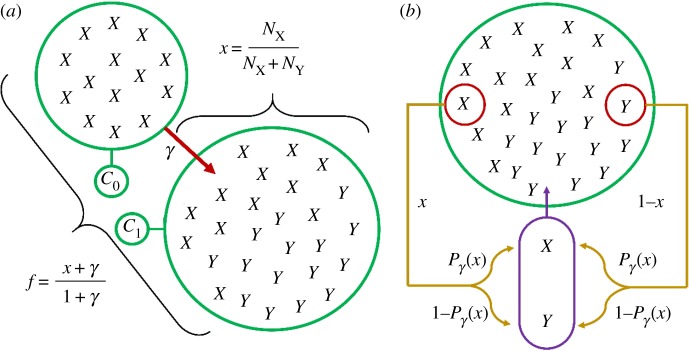
Schematic of model mechanisms. (*a*) Difference between absolute frequency *x* and relative frequency *f* in context *C*_1_. Absolute frequency *x* is given by the ratio of *X* occurrences encoded in *C*_1_. Effective frequency *f* also takes into account the *M* occurrences contained in the influential context *C*_0_, with a weight *γ* standing for the strength of this influence. (*b*) Schematic view of the process. At each iteration, either *X* or *Y* is chosen to be produced and thus encoded in memory, with respective probability *P*_*γ*_(*x*) and 1−*P*_*γ*_(*x*); the produced occurrence is represented here in the purple capsule. Another occurrence, already encoded in the memory, is uniformly chosen to be erased (red circle) so as to keep the population size constant. Hence the number of *X* occurrences, *N*_*X*_, either increases by 1 if *X* is produced and *Y* is erased, decreases by 1 if *Y* is produced and *X* is erased, or remains constant if the erased occurrence is the same as the one produced.

We want to make explicit the way *P*(*t*) depends on *x*(*t*), the absolute frequency of *X* in this context at time *t*. The simplest choice would be *P*(*t*)=*x*(*t*). However, we wish to take into account several facts. As context *C*_0_ exerts an influence on context *C*_1_, denoting by *γ* the strength of this influence (see electronic supplementary material, SIIB for an extended discussion on this parameter), we assume the probability *P* to rather depend on an effective frequency *f*(*t*) (figure 5*a*),

3.1 $$f(t)=\frac{N^{1}(t)+\gamma N^{0}(t)}{M+\gamma M}=\frac{x(t)+\gamma }{1+\gamma }.$$

We now specify the probability *P*(*f*) to select *X* at time *t* as a function of *f*=*f*(*t*). First, *P*(*f*) must be nonlinear. Otherwise, the change would occur with certainty as soon as the effective frequency *f* of the novelty is non-zero: That is, insofar as two meanings are related, the form expressing the former will also be recruited to express the latter. This change would also start quite abruptly, while sudden, instantaneous takeovers are not known to happen in language change. Second, one should preserve the symmetry between the two forms, that is, *P*(*f*)=1−*P*(1−*f*), as well as verify *P*(0)=0 and *P*(1)=1. Note that this symmetry is stated in terms of the effective frequency *f* instead of the actual frequency *x*, as production in one context always accounts for the contents of neighbouring ones.

For the numerical simulations, we made the following specific choice which satisfies these constraints:

3.2 $$P(f)=\frac{1}{2}\left\{1+\tanh\left(\beta \frac{f-(1-f)}{\sqrt{f(1-f)}}\right)\right\},$$

where *β* is a parameter governing the nonlinearity of the curve. Replacing *f* in terms of *x*, the probability to choose *X* is thus a function *P*_*γ*_(*x*) of the current absolute frequency *x*:

3.3 $$P_{\gamma }(x)=\frac{1}{2}\left\{1+\tanh\left(\beta \frac{2x-1+\gamma }{\sqrt{\left(x+\gamma )(1-x\right)}}\right)\right\}.$$

### 3.3. Analysis: bifurcation and latency time

The dynamics outlined above (figure 5*b*) is equivalent to a random walk on the segment [0;1] with a reflecting boundary at 0 and an absorbing one at 1, and with steps of size 1/*M*. The probability of going forwards at site *x* is equal to (1−*x*)*P*_*γ*_(*x*), and the probability of going backwards to *x*(1−*P*_*γ*_(*x*)).

For large *M*, a continuous, deterministic approximation of this random walk leads, after a rescaling of the time Mt→t, to a first-order differential equation for *x*(*t*):

3.4 $$\dot{x}=P_{\gamma }(x)-x.$$

**Figure 6.**
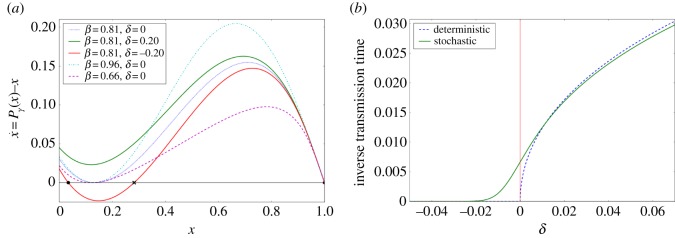
Evidence of a near-critical behaviour. (*a*) Speed $\dot{x}$ of the deterministic process for each of the sites, for different values of *β* and *δ*=(*γ*−*γ*_*c*_)/*γ*_*c*_, the distance to the threshold. Depending on the sign of *δ*, there is either one or three fixed points. (*b*) Inverse transmission time (time required for the system to go from 0 to 1), for the deterministic process (blue dotted line) and for the averaged stochastic process (green line), as a function of the control parameter *δ*. Deterministic transmission time diverges at the transition, while averaged stochastic transmission time remains finite.

This dynamics admits either one or three fixed points (figure 6*a*), *x*=1 always being one. Below a threshold value *γ*_*c*_, which depends on the nonlinearity parameter *β*, a saddle-node bifurcation occurs and two other fixed points appear close to a critical frequency *x*_*c*_. The system, starting from *x*=0, is then stuck at the smallest stable fixed point. The transmission time, i.e. the time required for the system to go from 0 to 1, becomes therefore infinite (figure 6*b*). Above the threshold value *γ*_*c*_, only the fixed point *x*=1 remains, so that the new variant eventually takes over the context for which it is competing. Our model thus describes how the strengthening of a cognitive link can trigger a semantic expansion process.

Slightly above the transition, a stranglehold region appears where the speed almost vanishes. Accordingly, the time spent in this region diverges. The frequency of the new variant will stick to low values for a long time, in a way similar to the latent behaviour evidenced by our dataset. This latency time in the process of change can thus be understood as a near-critical slowing down of the underlying dynamics.

Past this deterministic approximation, there is no more clear-cut transition (figure 6*b*) and the above explanation needs to be refined. The deterministic speed can be understood as a drift velocity of the Brownian motion on the [0;1] segment, so that in the region where the speed vanishes, the system does not move in average. In this region of vanishing drift, the frequency fluctuates over a small set of values and does not evolve significantly over time. Once it escapes this region, the drift velocity drives the process again, and the replacement process takes off. Latency time can thus be understood as a first-passage time out of a trapping region.

## 4. Numerical results

### 4.1. Model simulations

We ran 10 000 numerical simulations of the process described above (figure 5*b*), with the following choice of parameters: *β*=0.808, *δ*=0.0 and *M*=5000, where *δ*=(*γ*−*γ*_*c*_)/*γ*_*c*_ is the distance to the threshold. The specific value of *β* has been chosen to maximize *x*_*c*_. As *x*_*c*_ is the frequency at which the system gets stuck if *γ* is slightly below the threshold, it corresponds to the assumption that, even if the convention is not replaced, there is room for synonymic variation and the new variant can be used marginally. We chose *δ*=0.0 for the system to be purely diffusive in the vicinity of *x*_*c*_. The choice of *M* is arbitrary.

Even if this set of parameters remains the same throughout the different simulation runs, the quantities describing each of the 10 000 S-curves generated that way, especially the rate and the width, will change. It then becomes possible to obtain the statistical distributions of these quantities. Thus, while there is no one-to-one comparison between a single outcome of the numerical process and a given instance of change, we can discuss whether their statistical properties are the same.

**Figure 7.**
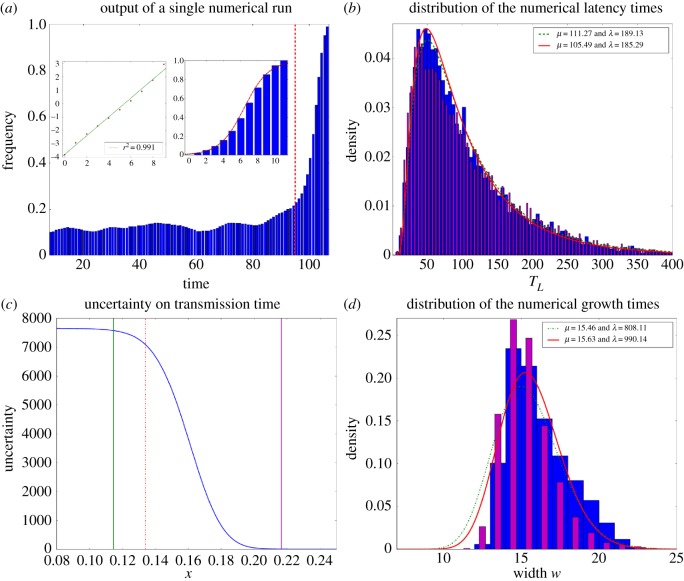
Numerical simulation of latency and growth times at the critical threshold. (*a*) Time evolution of the frequency of produced occurrences (output of a single run). Growth part and latency part are separated by a red dotted line. The logit transform (with linear fit) of the growth is shown in the left inset, alongside the sigmoidal fit of the rescaled frequency of the growth part (right inset). (*b*) and (*d*) Distribution of latency times (top) and growth times (bottom) over 10*k* processes, extracted from an empirical approach (blue wide histogram) and a first-passage time one (magenta thin histogram), with their respective inverse Gaussian fits (in red: empirical approach; in green: first-passage time approach). (*c*) Uncertainty on the transmission time given the position of the walker. The entrance and the exit of the trap are shown, respectively, by green and magenta lines. The red dotted line indicates the critical frequency *x*_*c*_. The trap corresponds to the region where the uncertainty drops from a high value to a low value.

From the model simulations, data are extracted and analysed in two parallel ways. On one side, simulations provide surrogate data: We can mimic the corpus data analysis and count how many tokens of the new variant are produced in a given time span (set equal to *M*), to be compared with the total number of tokens produced in this time span. We then extract ‘empirical’ latency and growth times (figure 7*a*), applying the same procedure as for the corpus data.

On the other side, for each run we track down the position of the walker, which is the frequency *x*(*t*) achieved by the new variant at time *t*. This allows to compute first-passage times. We then alternatively compute analytical latency and growth times (‘analytical’ to distinguish them from the former ‘empirical’ times) as follows. Latency time is here defined as the difference between the first-passage times at the exit and the entrance of a ‘trap’ region (see electronic supplementary material, SIB for additional details). Analytical growth time is defined as the remaining time of the process once this exit has been reached. Their distribution over 10 000 runs of the process are fitted with an inverse Gaussian distribution, which would be the expected distribution if the jump probabilities were homogeneous over the corresponding regions (an approximation then better suited for latency time than for growth time). Figure 7*b*,*d* shows the remarkable agreement between the ‘empirical’ and ‘analytical’ approaches, together with their fits by an inverse Gaussian distribution.

Crucially, those two macroscopic phenomena, latency and growth, are thus to be understood as of the same nature, which explains why their statistical distribution must be of the same kind. Furthermore, the boundaries of the trap region leading to the best correspondence between first-passage times and empirically determined latency and growth times are meaningful, as they correspond to the region where the uncertainty on the transmission time significantly decreases (figure 7*c*).

### 4.2. Confrontation with corpus data

**Figure 8.**
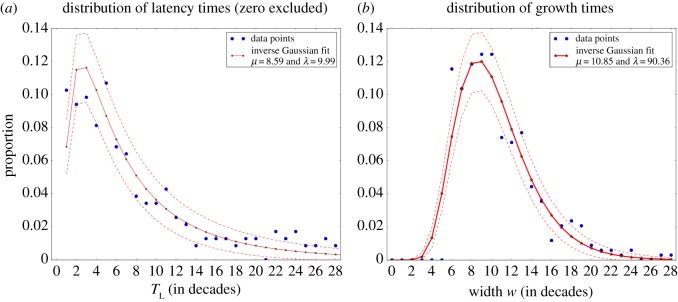
Inverse Gaussian fit of the latency times *T*_L_ (*a*) and the growth times *w* (*b*), extracted from corpus data. Data points are shown by blue dots, the inverse Gaussian fit being represented as a full red curve with star-shaped marks. The dashed red lines represent the standard deviation from the model. We detail in Materials and methods how we extracted these growth times and latency times from corpus data.

Our model predicts that both latency and growth times should be governed by the same kind of statistics, inverse Gaussian being a suited approximation of those. Inverse Gaussian distribution is governed by two parameters, its mean *μ* and a parameter *λ* given by the ratio *μ*^3^/*σ*^2^, *σ*^2^ being the variance. We thus try to fit the corpus data with an inverse Gaussian distribution (figure 8). In both cases, the Kullback–Leibler divergence between the data distribution and the inverse Gaussian fit is equal to 0.10. The rate *h* (slope of the logit) also follows a non-trivial distribution, as shown in electronic supplementary material, SIC.

Although there are short growth times in the frequency patterns of the forms we studied, below six decades they are not described by enough data points to assess reliably the specificity of the sigmoid fit. On figure 8 there are therefore no data for these growth times. The inverse Gaussian fit is not perfect, and is not expected to be: The model only predicts the distribution to be of the same family as the inverse Gaussian. Satisfyingly, among a set of usual distributions (exponential, Poisson, Gaussian, Maxwellian), the inverse Gaussian proves to be the most adequate for both the growth and the latency (see electronic supplementary material, SIC for additional details).

The main quantitative features extracted from the dataset are thus correctly mirrored by the behaviour of our model. We confronted the model with the data on other quantities, such as the correlation between growth time and latency time, two quantities which our model predicts to be independent. There again, the model proves to match appropriately these quantitative aspects of semantic expansion processes (see electronic supplementary material, SID).

## 5. Discussion

Based on a corpus-based analysis of frequency of use, we have established two robust stylized facts of semantic change: an S-curve of frequency growth, already evidenced in the literature, and a preceding latency period during which the frequency remains more or less constant, typically at a low value. We have proposed a model predicting that these two features, albeit qualitatively quite different, are two aspects of one and the same phenomenon.

Our analysis is based on the *a priori* assumption that a frequency rise is caused by a semantic expansion. An alternative would be the reverse mechanism, that semantic expansion is induced by an increase in the frequency of use. Actually, it is not infrequent to find unambiguous traces of the semantic expansion throughout and even before the latency phase. Also, we often looked for forms in a syntactic context compatible only with the new meaning—e.g. for *j’imagine* we searched specific intransitive patterns, like ‘il y a de quoi, j’imagine, les faire étrangler’ (1783) (There’s good reason to have them strangled, I suppose)—so that, in such cases, it leaves no doubt that the latency phase and the frequency rise are posterior to the semantic expansion. The model, however, does not exclude that both mechanisms are at work, as discussed in electronic supplementary material, SIIB.

The detailed hypotheses on which our model lies are well grounded on claims from Cognitive Linguistics: Language is resilient to change (nonlinearity of the *P* function); language users have cognitive limitations; the semantic territory is organized as a network whose neighbouring sites are asymmetrically influencing each other. The overall agreement with empirical data tends to suggest that language change may indeed be cognitively driven by semantic bridges of different kinds between the concepts of the mind, and constrained by the mnemonic limitations of this very same mind.

According to our model, the onset of change depends on the strength of the conceptual link between the source context and the target context: If the link is strong enough, that is, above a given threshold, it serves as a channel so that a form can ‘invade’ the target context and then oust the previously established form. In a sense, the sole existence of this cognitive mapping is already a semantic expansion of some sort, yet not necessarily translated into linguistic use. Latency is specifically understood as resulting from a near-critical behaviour: If the link is barely strong enough for the change to take off, then the channel becomes extremely tight and the invasion process slows down drastically. These narrow channels are likely to be found between lexical and grammatical meanings [33,34]. This would explain why the latency-growth pattern is much more prominent in the processes of grammaticalization, positing latency as a phenomenological hint of this latter category.

As acknowledged by a few authors [35,36], it is interesting to note that, in the literature, the S-growth is given two very different interpretations. According to the first one, an S-curve describes the spread of the novelty in a community of speakers [4,37–39]; as for the second one, it reflects the spread in language itself, the new variant being used in an increasing number of contexts [17,40–42]. According to the interpretation we give to our model, the diffusion chiefly happens over the linguistic memory of the whole speech community. It does not involve some binary conversion of individuals towards the new variant; it is a spread within the individuals rather than a spread among them. On the other hand, the S-curve arises in the taking over of a single context, and does not rely on a further diffusion over additional contexts to appear. Though the latter spread need thus not be responsible for the S-shape, it may nonetheless influence the evolution in other ways (e.g. the total duration). The interplay between the specific features of an S-curve and the structure of the conceptual network remains to be investigated.

We note, however, that our model may be given a different, purely sociolinguistic interpretation, as discussed in electronic supplementary material, SIIC. Nevertheless, several arguments argue against this interpretation. First, the semantic evolution involves very long timescales, up to several centuries [41]; second, societal diffusion, of a new technological device, for instance, is associated with a specific scaling law between the steep and duration of the S-curve of $-\frac{2}{3}$ [43], which is very different from the behaviour of the forms in our dataset, where no scaling law is to be found (the two parameters are related by a trivial −1.0 exponent; see electronic supplementary material, SID).

Recently, the nature of linguistic change has been investigated through different case studies, separating internal (imitation between members of a community) and external (e.g. linguistic reforms from language academies) factors of change [21]. While internal factors give rise to an S-curve, external factors lead to an exponential growth of frequency; hence, the S-curve is not the only dynamics by which language change can occur. However, in this work, agents choose between the two variants on a binary basis, and language-based mechanisms, such as the network asymmetric links at the core of our own model, would count as an external mechanism. These strong differences make it difficult to quantitatively compare their approach and ours, although it is to be agreed that S-curves contain crucial information on language change and need to be investigated and quantified further on. Moreover, as semantic change is seldom driven by external forces such as linguistic reforms, the exponential pattern is not to be expected in this case, and indeed we have not found it in our dataset.

Finally, we argue that our results, though grounded on instances of semantic expansion in French, apply to semantic expansion in general. The time period covered is long enough (700 years) to exclude the possibility that our results be ascribable to a specific historical, sociological or cultural context. The French language itself has evolved, so that Middle French and contemporary French could be considered as two different languages, yet our analysis applies to both indistinctly. Besides, the latency-growth pattern is to be found in other languages; for instance, although Google Ngram cannot be used here for a systematic quantitative study, specific queries for constructions such as *way too*, *save for*, *no matter what*, yield qualitative frequency profiles consistent with our claims. Our model also tends to confirm the genericity of this pattern, as it relies on cognitive mechanisms whose universality has been well evidenced [44,45].

## 6. Material and methods

### 6.1. Corpus data

We worked on the *Frantext* corpus [22], which in 2016 contained 4674 texts and 232 millions of words for the chosen time range. More details are given in electronic supplementary material, SIIIB. It would have been tempting to make use of the large database Google Ngram, yet it was not deemed appropriate for our study, as we explain in electronic supplementary material, SIIIC.

We studied changes in frequency of use for about 400 instances of semantic expansion processes in French, on a time range going from 1321 up to the present day. See electronic supplementary material, SIIID for a complete list of the studied forms.

### 6.2. Extracting patterns from corpus data

#### 6.2.1. Measuring frequencies

We divided our corpus into 70 decades. Then, for each form, we recorded the number of occurrences per decade, dividing this number by the total number of occurrences in the database for that decade. The output number is called here the *frequency* of the form for the decade, and is noted *x*_*i*_ for decade *i*. To smooth the obtained data, we replaced *x*_*i*_ by a moving average, that is, for *i*≥*i*_0_+4, *i*_0_ being the first decade of our corpus: $x_{i}\leftarrow \frac{1}{5}\sum_{k=i-4}^{i}x_{k}.$

#### 6.2.2. Sigmoids

We looked for major increases in frequency. When such a major shift is encountered, we automatically (see below) identify frequencies $x_{\min}$ and $x_{\max}$, respectively, at the beginning and the end of the increasing period. If we, respectively, note *i*_start_ and *i*_end_ the decades for which $x_{\min}$ and $x_{\max}$ are reached, then we define the width (or growth time) *w* of the increasing period as *w*=*i*_end_−*i*_start_+1. To quantify the sigmoidal nature of this growth pattern, we apply the logit transformation to the frequency points between $x_{\min}$ and $x_{\max}$:

6.1 $$y_{i}=\log\left(\frac{x_{i}-x_{\min}}{x_{\max}-x_{i}}\right).$$

If the process follows a sigmoid ${\tilde{x}}_{i}$ of equation

6.2 $${\tilde{x}}_{i}=x_{\min}+\frac{x_{\max}-x_{\min}}{1+e^{-hi-b}},$$

then the logit transform of this sigmoid satisfies ${\tilde{y}}_{i}=hi+b$. We thus fit the *y*_*i*_’s given by (6.1) with a linear function, which gives the slope (or rate) *h* associated with it and the residual *r*^2^ quantifying the quality of the fit. The boundaries *i*_start_ and *i*_end_ have been chosen so as to maximize *w*, with the constraint that the *r*^2^ of the linear fit should be at least equal to a value depending on the number of points, in order to ensure that the criterion has a *p*-value significance of less than 0.05 according to a null model of frequency growth. Further explanations are provided in electronic supplementary material, SIA.

#### 6.2.3. Latency period

In most cases (69% of sigmoidal growths), one observes that the fast increasing part is preceded by a phase during which the frequency remains constant or nearly constant. The duration of this part, denoted by *T*_L_ (latency time) in this paper, is identified automatically as follows. Starting from the decade *i*_start_, previous decades *j* are included in the latency period as long as they verify $|x_{j}-x_{\min}|<0.15*(x_{\max}-x_{\min})$ and *x*_*j*_>0, and cease to be included either as soon as the first condition is not verified, or if the second condition does not hold for a period longer than 5 decades. Then the start *i*_lat_ of the latency point is defined as the lowest *j* verifying both conditions, so that *T*_L_ is given by *T*_L_=*i*_start_−*i*_lat_.

## Supplementary Material

Supplementary_material

## Supplementary Material

full_data.zip

## Data accessibility

The datasets supporting this article have been uploaded as part of the electronic supplementary material (see electronic supplementary material, SIIIA).
