# Supplementary material for: Frequency patterns of semantic change: corpus-based evidence of a near-critical dynamics in language change
Source: R Soc Open Sci. 2017 Nov 8;4(11):170830. doi: 10.1098/rsos.170830 (PMC5717648; doi:10.1098/rsos.170830)
Supplement: Supplementary_material [file rsos170830supp1.pdf]

Frequency patterns of semantic change:
Corpus-based evidence of a near-critical dynamics in language change

Feltgen Quentin, Fagard Benjamin and Nadal Jean-Pierre

Supplementary material

CONTENTS

I. Further data analysis 2
A. Null model of frequency growth and significance of the sigmoidal fit 2
1. Stochastic null model 2
2. Alternative null models 3
3. Robustness of the sigmoidal fit 4
B. Boundaries of the trap region 5
1. Analytical computation of mean first passage times 5
2. Trap boundaries 6
C. Statistical distributions 7
1. Growth time 7
2. Latency time 8
3. Statistical distribution of the slopes 10
D. Further comparisons with corpus data 11
1. Péclet number 11
2. Growth-Slope correlation and scaling law 13
3. Latency-Growth correlation 14

II. Model variants 15
A. Hearer mechanism 15
1. Hearer variant 15
2. Combined model 17
3. Summary 17
B. Interpretations of the cognitive strength γ 17
1. Heterogeneous memory sizes 18
2. Different probabilities of use 18
C. Sociolinguistic interpretation 19

III. Corpus data 20
A. Raw data 20
B. Frantext textual database 20
A note on French 22
C. Why not using Google Ngram? 23
D. Studied forms 25

References 40

I. FURTHER DATA ANALYSIS

A. Null model of frequency growth and significance of the sigmoidal fit

To evaluate the significance of the sigmoidal fit, we need to compare it with a null model of frequency growth. However, what would be the null hypothesis in this case is far from obvious. Given that the frequency has risen from x_{min} to x_{max} in a time w, which model of growth would be the closest to an assumption-free one? As the frequency can be rescaled using the following formula:

$$x \leftarrow \frac{x - x_{min}}{x_{max} - x_{min}} \,, \tag{1}$$

the matter can be simplified by considering a growth from 0 to 1.

1. *Stochastic null model*

A simple choice is to consider the following random walk, with Gaussian jumps at each time step:

$$x_{t+1} = x_t + \frac{1}{w}(1 + \eta_t) \,, \tag{2}$$

where η_t is a random term drawn from a normal distribution of mean 0 and variance 1, with the initial condition $x_0 = 0$. The mean process would be a linear growth from 0 to 1 with w steps of size $1/w$.

In the main text, we extracted the S-curve according to the following procedure:

- search for all pairs t_{min} and t_{max}, with $t_{max} - t_{min} > 5$, so that the logit transform of the data points x_t in-between is associated with a linear fit of sufficiently good quality
- retain only the pairs associated with the greatest possible width w ($w = t_{max} - t_{min} + 1$);
- select among those ones the pair with the best r^2 coefficient of the linear fit of the logit.

The question is then: what is a linear fit of sufficiently good quality? Now that we have a null model, we can devise a criterion $r^2_{min}(w)$ so that the fit is associated with a p-value below 0.05: if the r^2 of the fit is higher than this criterion, then the sigmoidal fit is deemed significant.

To do so, for a given value of w, we generated $50,000$ growth processes and computed the ratio p of processes obeying the criterion. This ratio gives thus the p-value associated with the criterion. The criterion was then increased so as to pass below the threshold $p < 0.05$ (Fig. 1). The same can be done for any threshold of significance (e.g. $p < 0.001$). As can be seen from Fig. 1, a very high criterion must be set to insure significance for low number of points (a width of T is associated with $T - 2$ points). The criterion is non-monotonic and increases for large number of points. Indeed, in these cases, the noise $1/w$ becomes weak and the process tends to a linear curve, which can be easily compatible with a sigmoid. In our data survey, we used the criterions associated with the $p < 0.05$ threshold of significance.

2. *Alternative null models*

We could have used other null models. A possibility we investigated is the following. We posit a saturating growth function $G(x)$ given by :

$$G(x) = \left[1 - (x-1)^{2n}\right]^{1/2n} , \tag{3}$$

where n is a positive integer. This insures an infinite derivative at $x = 0$ and a null derivative at $x = 1$: the process can start as quickly and end as slowly as one wishes. The outcome weakly depends on this parameter n, which can be set to 1. Then, the null process of growth would be as follows:

$$x_{t+1} = x_t + \eta_t , \tag{4}$$

where η_t is drawn from the distribution :

$$P(\eta_t, x_t, t)) = \frac{1}{Z} \exp\left\{ -\frac{\lambda(\eta_t - x_t)}{G(t/w) - x_t} \right\} , \tag{5}$$

with λ a parameter that we set to 5.

This model allows for a wider diversity of processes (there can be sudden jumps), but can hardly be qualified as a null hypothesis. Also, it enforces a strict monotony, which is frequent in the data, but not necessary. Nonetheless, it gave rise to criterions close to those

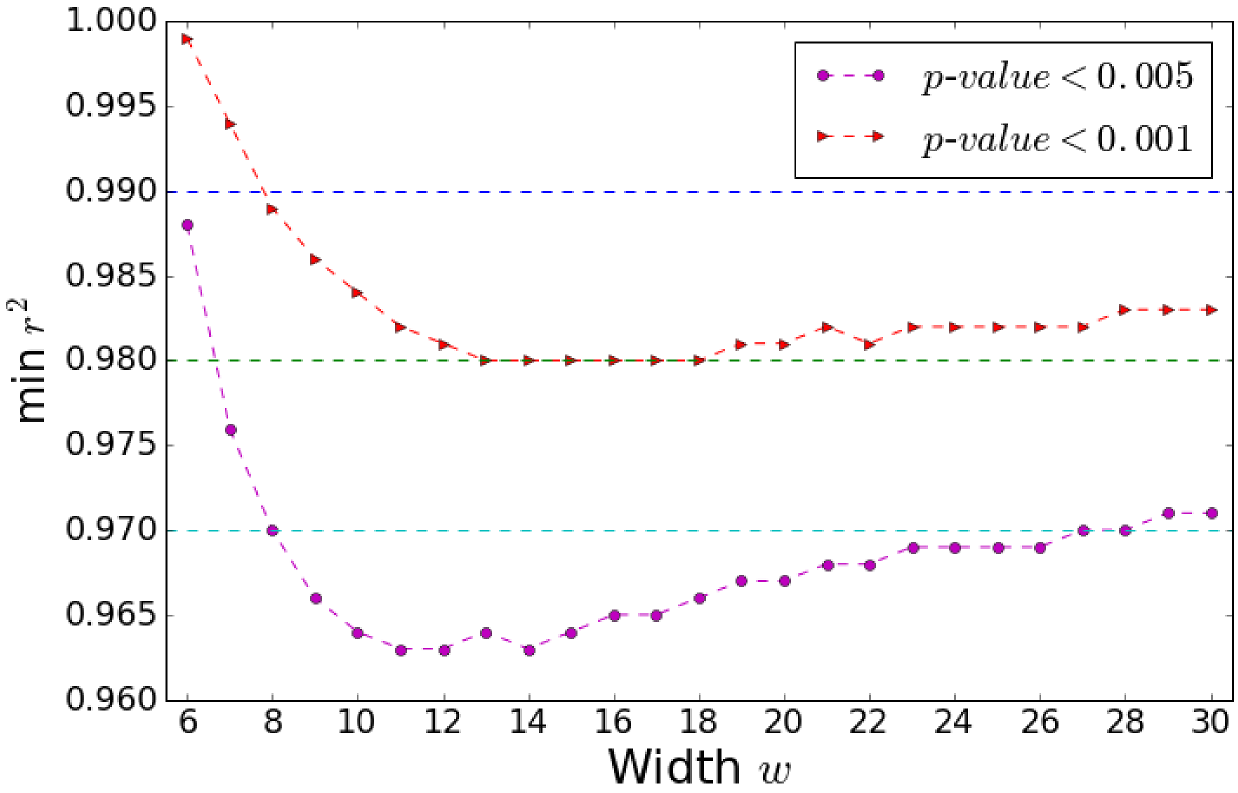

FIG. 1. Minimal quality of the linear fit of the logit transform so as to insure the significance of a sigmoidal fit of the data as compared to a random null model of frequency growth.

found in the preceding null model. As a conclusion, we can only stress that a null model of growth is already an assumption of some sort, and it is unclear how much theoretical a priori is feeding the null hypothesis.

3. Robustness of the sigmoidal fit

We can alternatively address the statistical robustness of the sigmoidal fit. To do so, we compute, for each point, the expected fluctuation that the sigmoidal model would predict for a finite sample size associated with the number of occurrences $\tilde{N}_t$ characterizing decade. t We make use of the standard confidence interval of 95% probability:

$$\tilde{n}_t = \tilde{x}_t \tilde{N}_t \pm 1.96\sqrt{\tilde{x}_t(1-\tilde{x}_t)\tilde{N}_t}\,, \tag{6}$$

where $\tilde{n}_t$ is the expected number of occurrences, and $\tilde{x}_t$ the probability of the form to be produced, according to the sigmoidal fit:

$$\tilde{x}_t(h, b, x_{min}, x_{max}) = x_{min} + \frac{x_{max} - x_{min}}{1 + e^{-ht-b}}\,. \tag{7}$$

Therefore, the actual number x_t of occurrences must obey, for the sigmoidal fit $\tilde{x}_t$ to be consistent with the data:

$$\tilde{x}_t - 1.96\sqrt{\frac{\tilde{x}_t(1-\tilde{x}_t)}{\tilde{N}_t}} < x_t < \tilde{x}_t + 1.96\sqrt{\frac{\tilde{x}_t(1-\tilde{x}_t)}{\tilde{N}_t}}\,,\ \forall t \in [t_{start} : t_{end}], \tag{8}$$

where t_{start} and t_{end} are the time boundaries of the extracted pattern, respectively associated with frequencies x_{min} and x_{max}.

Note that, as the data x_t is a gliding average of the frequency, the number of occurrences of decade t is not straightforwardly given by the number N_t of occurrences in the corpus. This is why we made use in the above formulae of an 'effective' number of occurrences associated with decade t, $\tilde{N}_t$, given by:

$$\tilde{N}_t = \frac{1}{W}\sum k = t - W + 1^t N_k\,. \tag{9}$$

Another remark to be made is that these expected fluctuations are due to the finite size of the sample. Other sources of fluctuations are nonetheless to be expected, such as inhomogeneities in the sample (e.g. if the linguistic data in the corpus is dominated by a handful of authors). Therefore, fluctuations in equation (6) should be considered as lower bounds for the true fluctuations, which we cannot know precisely.

The robustness of all sigmoidal patterns extracted from our data have therefore been checked through equation (8). The result of this check, for each pattern, has been reported on the Table of all studied forms (section III D). For 292 patterns out of the 338 extracted (approximately 86% of the total), all data points lie within the confidence interval (Fig. 2a), which proves that the data is consistent with the sigmoidal fit. For the remaining 46 patterns, one or several datapoints lied outside the confidence interval (Fig. 2b). As the fluctuations are underestimated, we did not withdraw these patterns from the computation of the statistical patterns. This test serves only to assess the consistency of at least 86 % of the sigmoidal patterns, supporting our claim that the present statistical analysis confirms the robustness of the S-curve agreed on in the literature.

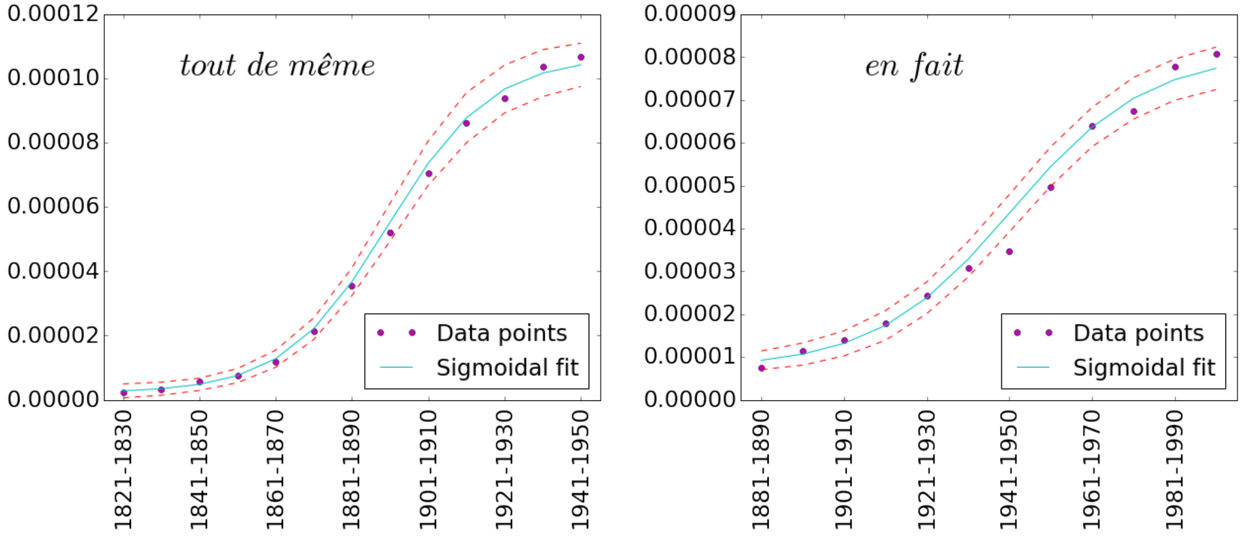

(a) (b)

FIG. 2. Frequency data (magenta dots) associated with the extracted pattern from (a) *tout de même* and (b) *en fait*. The sigmoidal model is shown in cyan and the associated confidence interval is shown in red dashed lines. All datapoints lie within this confidence interval in the first case (*tout de même*). In the second case (*en fait*), datapoints associated with decades 1941-1950 and 1951-1960 lie outside the interval. Therefore, the S-curve may not be a reliable fit of the data.

B. Boundaries of the trap region

The analytical definitions, used to compute the latency and growth times in the model, are based on first passage times. In this section we outline the procedure we followed to compute these times.

1. *Analytical computation of mean first passage times*

Let us note $T_{n\to m}$ the first passage time at site m, starting at site n, $0 \leq n,\ m \leq M$. This is a random variable for which one can write down a recursion equation for its generatrix function:

$$\left\langle e^{\lambda T_{n\to m}} \right\rangle = R_n \left\langle e^{\lambda (T_{n+1\to m}+1)} \right\rangle + L_n \left\langle e^{\lambda (T_{n-1\to m}+1)} \right\rangle + (1 - L_n - R_n) \left\langle e^{\lambda (T_{n\to m}+1)} \right\rangle , \qquad (10)$$

where R_n and L_n are, respectively, the forward and backward jump probabilities, and $\langle . \rangle$ denotes the average. We recall that $n = 0$ is a reflecting boundary ($L_0 = 0, R_0 > 0$), and $n = M$ an absorbing boundary ($R_M = L_M = 0$). We have $T_{n\to n} = 0$, and for the left boundary condition, that is for $n = 0$:

$$\left\langle e^{\lambda T_{0\to m}} \right\rangle = R_0 \left\langle e^{\lambda (T_{1\to m}+1)} \right\rangle + (1 - R_0) \left\langle e^{\lambda (T_{0\to m}+1)} \right\rangle . \qquad (11)$$

The first and second derivatives of equation (10) with respect to λ leads for $\lambda = 0$ to recurrence relations for the first and second moment of $T_{n\to m}$, respectively.

More specifically, we can compute the first two moments of the first passage time between one site and its immediate successor, $T_{i\rightarrow i+1}$:

$$\langle T_{i\rightarrow i+1}\rangle = t_i \tag{12}$$

And:

$$\left\langle T^2_{i\rightarrow i+1}\right\rangle = u_i\,, \tag{13}$$

Where the t_i's and u_i's are iteratively computed from:

$$\begin{cases} t_0 = \dfrac{1}{R_0} \\ \\ u_0 = \dfrac{2t_0 - 1}{R_0} \end{cases} \tag{14}$$

And:

$$\begin{cases} t_i = \dfrac{1}{R_i} + \dfrac{L_i}{R_i} t_{i-1} \\ \\ u_i = 2t_i^2 + \dfrac{L_i}{R_i} u_{i-1} \end{cases} . \tag{15}$$

From this, we can easily compute the first two moments for any $T_{n\rightarrow m}$:

$$\mu\left(T_{n\rightarrow m}\right) = \sum_{k=n}^{m-1} t_k \tag{16}$$

And:

$$\sigma^2\left(T_{n\rightarrow m}\right) = \sum_{k=n}^{m-1}\left(u_k - t_k^2\right) \tag{17}$$

2. Trap boundaries

In the main text, we explain latency time and growth time as first passage times. However, these two quantities are both empirically extracted from the macroscopic pattern obtained at the end of a run, in a procedure exactly transposed from the corpus data treatment. The question is then: Which trap boundaries n_{in} and n_{out} should we set in order for the properly defined time $T_{n_{in}\rightarrow n_{out}}$ to correspond statistically to the empirically defined latency time?

Besides, growth time can be seen as well as a first passage time between two sites. Though the exit site should be M, it is more appropriate to define a cut-off n_{last}. Indeed, there is a discrepancy between the fact that, close to the absorbing point, the walk gets slowed down again, and that, in this region, the new variant is almost always produced anyway. In other terms, growth time, as extracted from the time evolution of the ratio of produced new variant occurrences, is not sensitive whether the end of the walk is reached or not.

Let us note μ_g and σ_g^2, and μ_{lat} and σ_{lat}^2, respectively the mean and the variance of the growth and latency times (obtained from the distributions of those empirically extracted quantities from ten thousand runs). Then, over a reasonable range of n, we look for m so that $\mu\left(T_{n\rightarrow m}\right)$ is as close as possible to μ_g; we then choose the pair $(n; m)$ such that $\sigma^2\left(T_{n\rightarrow m}\right)$

TABLE I. Output of three statistical tests (Kullback-Leibler divergence (D_{KL}), Akaike Information Criterion (AIC) and Bayesian information Criterion (BIC), to compare different fits of the growth times distribution.

Test	Poisson	Maxwellian	Gaussian	Inverse Gaussian
D_{KL}	0.21	0.26	0.35	0.10
AIC	227	259	323	152
BIC	231	263	331	160

is as close as possible to σ_g^2. This pair defines thus the region of growth, $(n_{out}; n_{last})$. We then choose n_{in} so as to fit the mode of the empirical latency distribution, assuming that first passage time is distributed according to an Inverse Gaussian (which entails that the mode is a known function of μ and σ^2).

C. Statistical distributions

In the main paper, we presented the statistical distributions of both the latency times and the growth times obtained from corpus data, and proposed an Inverse Gaussian fit of the result, following the theoretical prediction that the distribution should be of the same family as the Inverse Gaussian. We can now consider whether other usual statistical distributions could be suited as well to account for the statistical features of our dataset.

1. *Growth time*

We tried to fit the distribution of growth times with three different usual statistical distributions: Poisson, Maxwellian, and Gaussian (Fig 3). Aside from the Poisson distribution, the fit is qualitatively inadequate compared to an Inverse Gaussian fit.

We can further assess which of these four trials is to be favored by computing the Kullback-Leibler divergence between these theoretical proposals and the corpus data. We remind that the Kullback-Leibler divergence is closely related to the likelihood, and maximizing the likelihood is strictly equivalent to minimizing the Kullback-Leibler divergence. We obtained Kullback-Leibler divergences of 0.21, 0.26, 0.35 and 0.10 for the Poisson, Maxwellian, Gaussian and Inverse Gaussian distributions, respectively. Other statistical tests have been performed to account for the difference in the number of parameters between these distributions (1 for Poisson vs. 2 for the three others) and reported on Table I. Therefore, even if the Poisson distribution seems adequate, it does not perform much better than the Maxwellian. This failure is imputable to the tail of the distribution, which is thicker than what a Poisson distribution would predict. This tail is captured by the Maxwellian, but the latter distribution fails to reproduce the peak of the distribution.

Comparatively, the Inverse Gaussian fit is significantly better than the other three. It is adequate for both the peak and the tail. Therefore, albeit the data is not perfectly fit by the Inverse Gaussian, this distribution displays the right behavior, as we predicted from our model.

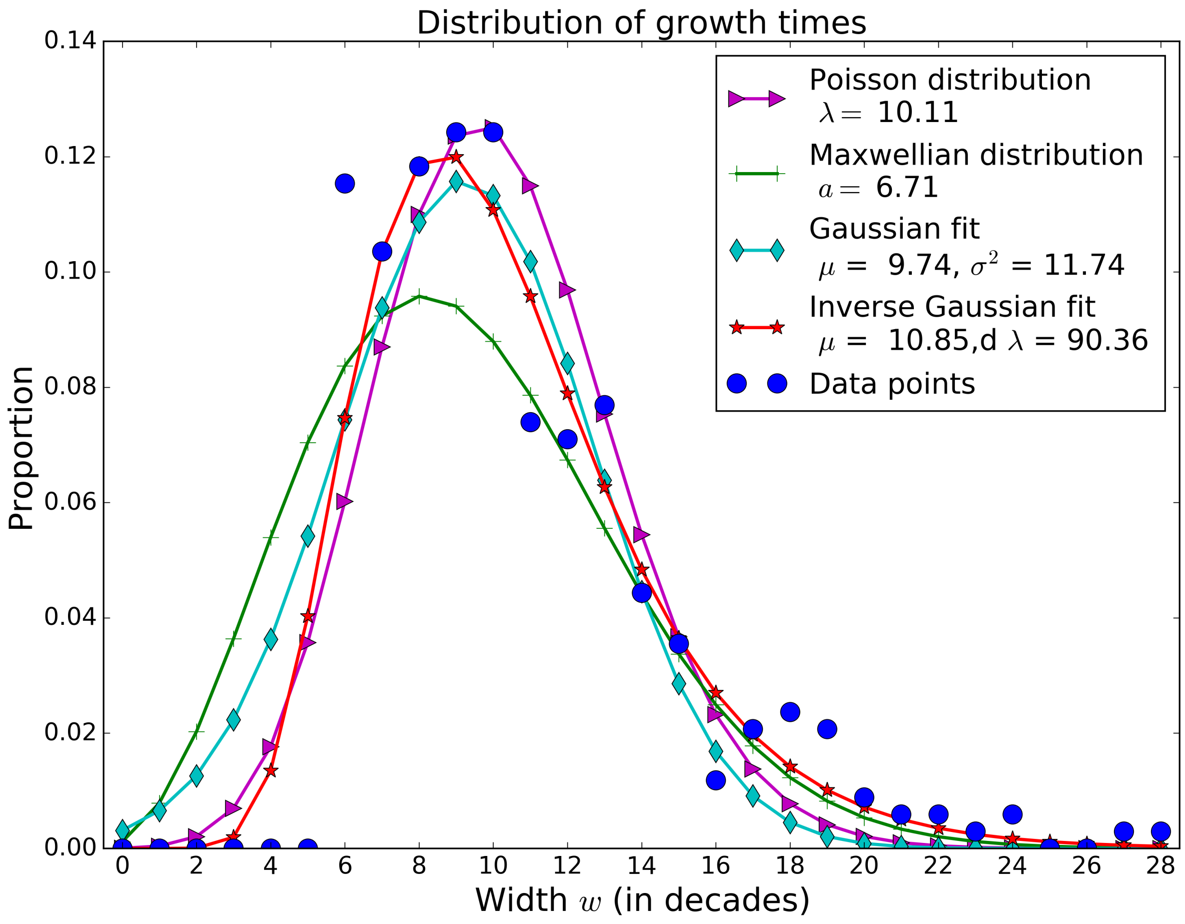

FIG. 3. Several fits of the distribution of growth times as extracted from corpus data.

2. Latency time

We can do the same for the distribution of latency times. We tried, besides the Inverse Gaussian, the exponential and the Gaussian distributions, as the Maxwellian and the Poisson distributions were largely inadequate (Fig. 4).

The same statistical tests as before have been performed to select the best distribution (Table II). Once more the Inverse Gaussian proves to be superior, even though the exponential also displays the right qualitative behavior. Also, we can compare the parameters obtained from an optimization fit with the actual mean of the data, which is 8.59. The mean should be given by the parameter a of the exponential and the parameter μ of both the Gaussian and the Inverse Gaussian. In this regard, it is clear that the Gaussian can be ruled out (it predicts a mean of 4.29) while the exponential and the Inverse Gaussian are consistent with the data (they respectively predict a mean of 7.06 and 9.72). An interesting difference between the exponential distribution and the Inverse Gaussian one would be that the mode of the distribution is zero in the former case, and non-zero in the latter. This feature could be further investigated with a larger amount of data regarding the latency, so as to clarify the behavior of the distribution in the region of lower values of the latency time. A finer timescale would also allow to zoom in this region of low latency times, so as

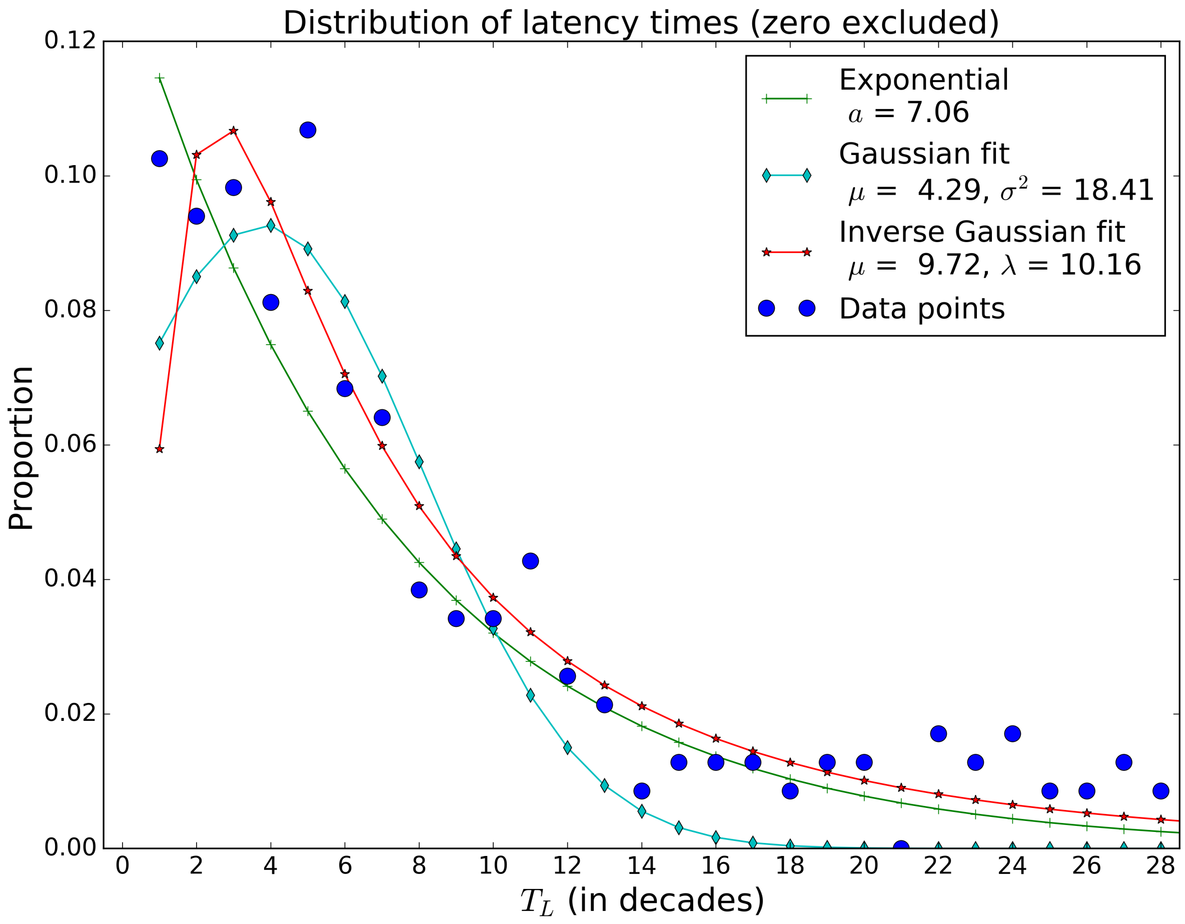

FIG. 4. Several fits of the distribution of latency times as extracted from corpus data.

TABLE II. Output of three statistical tests (Kullback-Leibler divergence (D_{KL}), Akaike Information Criterion (AIC) and Bayesian information Criterion (BIC), to compare different fits of the latency times distribution.

Test	Exponential	Gaussian	Inverse Gaussian
D_{KL}	0.24	1.71	0.10
AIC	834	1393	717
BIC	837	1400	725

to investigate whether the behavior of the distribution is non-monotonic in this domain, as would predict the Inverse Gaussian.

Here again, the Inverse Gaussian appears to capture more closely the corpus data than the other usual statistical distributions, as predicted from the model. It is also worth noticing that the model predicts that the Inverse Gaussian would be suited for both the growth and the latency, while the other candidates are appropriate for only one of these quantities (the growth time for the Poisson distribution, the latency time for the exponential).

TABLE III. Output of three statistical tests (Kullback-Leibler divergence (D_{KL}), Akaike Information Criterion (AIC) and Bayesian information Criterion (BIC), to compare different fits of the slopes distribution.

Test	Maxwellian	Gaussian	Inverse Gaussian	Scaling law fit
D_{KL}	0.14	0.20	0.10	0.14
AIC	243	293	222	252
BIC	240	285	215	244

3. Statistical distribution of the slopes

From the empirical procedure, we can also extract, for both the corpus and numerical datasets, the statistical distributions of the slopes of the logit transform of the sigmoidal part. Corpus data (Fig. 5) is best fitted by the Inverse Gaussian, by comparison with a Maxwellian and a Gaussian. Statistical tests favor consistently the Inverse Gaussian (Table III). All these three fits have been done without optimization, using the mean and the variance of the data to compute the parameters accordingly.

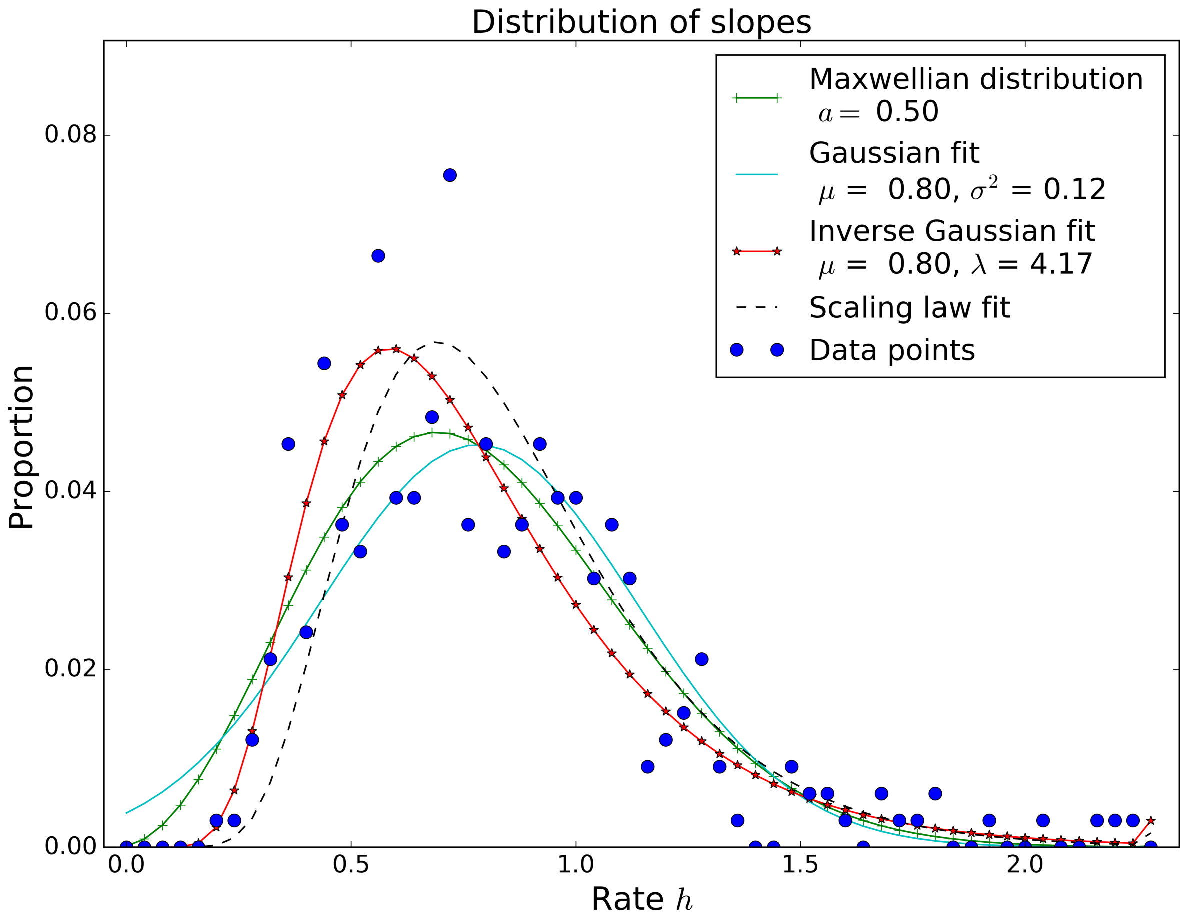

FIG. 5. Several fits of the distribution of the slopes as extracted from corpus data.

Why the distribution of the slopes would follow an Inverse Gaussian is unclear though. From the scaling relation between the slope and the width (see section I D), we can derive that the slopes h must be distributed according to the density ρ_h given by:

$$\rho_h(h) = \frac{e^{2.10}}{h^2}\rho_w\left(\frac{e^{2.10}}{h}\right) . \tag{18}$$

Assuming for ρ_w an Inverse Gaussian with parameters obtained from the Inverse Gaussian fit of the corpus data for the growth time, we can propose an estimate of the statistical distribution for the slopes. As can be seen on Fig. 5, this curve is qualitatively appropriate, hinting therefore at the consistency between our different results. The associated Kullback-Leibler divergence is equal to 0.14, the same as for the Maxwellian, not far from an Inverse Gaussian fit.

There is another prediction that we can make regarding this matter. If we assume that the growth is Inverse Gaussian, then according to the scaling law relating the width w and the slope h (see section I D):

$$h \approx \frac{e^{2,10}}{w} , \tag{19}$$

we can predict that, under the assumption that the width is Inverse Gaussian distributed:

$$\begin{aligned}\langle h\rangle &\approx e^{2.10}\left(\frac{1}{\mu_w}+\frac{1}{\lambda_w}\right)\\ &\approx e^{2,10}\left(\frac{1}{10.85}+\frac{1}{90.36}\right)\\ &\approx 0.84\end{aligned} \tag{20}$$

which is close to what we find in the data ($\langle h\rangle = 0.80$).

On the other hand, the distribution of the slopes generated from numerical data is best fitted by a Gaussian (Fig. 6), with a Kullback-Leibler divergence of 0.009 compared to 0.013 for the Inverse Gaussian.

This may be explained by the fact that an Inverse Gaussian distribution tends to a Gaussian one whenever parameter λ tends to infinity. The fact that λ is much bigger compared to μ in numerical data than in corpus data implies that there are more sources of variation for the growth part of the process in the data than what we considered in the model. We discuss this issue in the next subsection.

D. Further comparisons with corpus data

In our paper, we show that an Inverse Gaussian distribution is adequate to capture both latency time and growth time distributions, indicating that these two quantities are of the same nature, and result from the same mechanism of change. However, the agreement between our model and the corpus data goes further, as we show in this section.

1. *Péclet number*

The parameters μ and λ of the Inverse Gaussian distribution scale with the time length in the same way, so that is is relevant to consider their ratio, which is called the Péclet

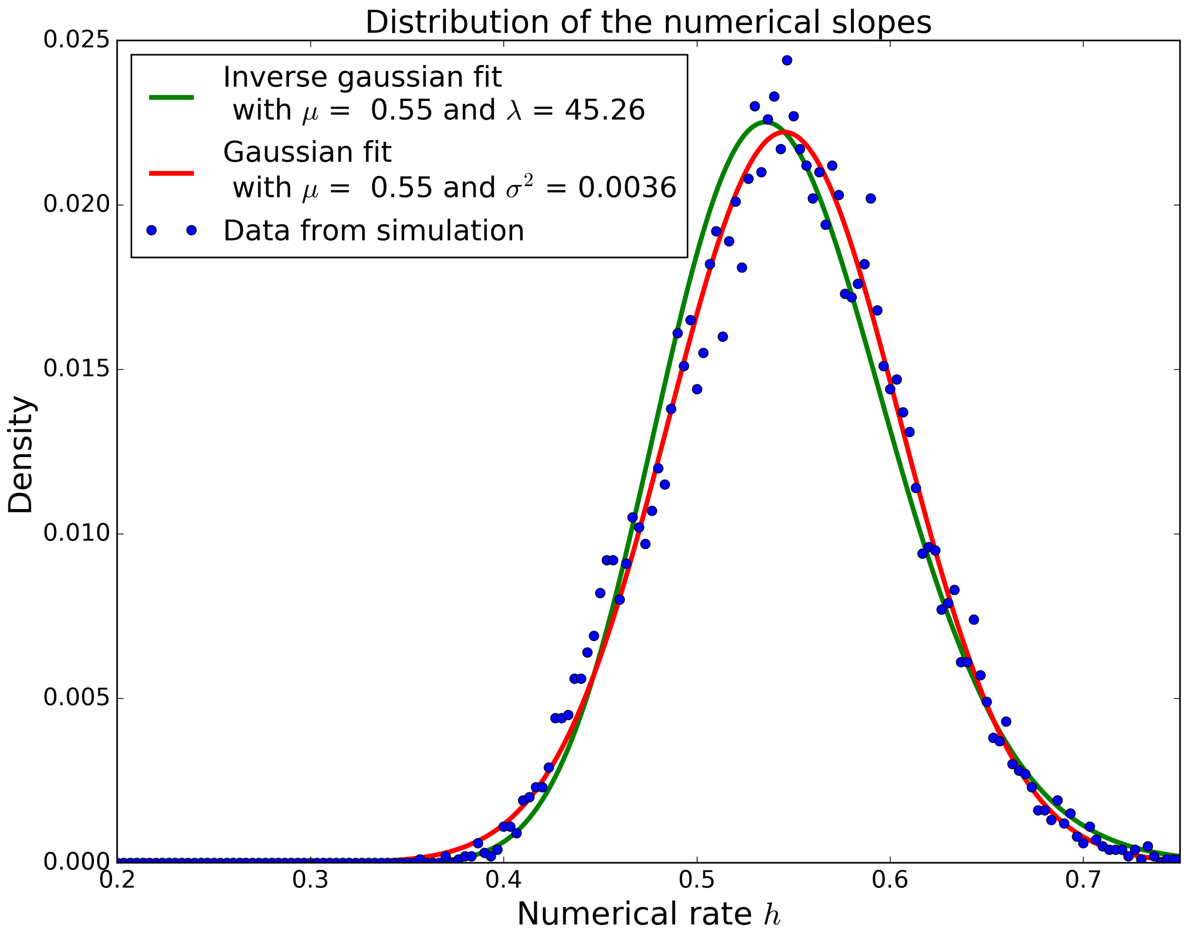

FIG. 6. Inverse Gaussian and Gaussian fits of the distribution of slopes as extracted from numerical data.

number [1]. Note that, because the relation $\lambda = \mu^3/\sigma^2$ holds, the Péclet number is but the ratio between the squared mean and the variance.

The Péclet number for latency times from corpus data is equal to 2.3 while the model gives back a Péclet number of 1.4, so they both are of the same order of magnitude. However, for growth times, we get 10.4 for corpus data, and 63 in the model, so that there is no agreement between the two.

Actually, this discrepancy is rather expected. Given the definition of the Péclet number, it means that the variance of the growth time is comparatively greater in the data than it is in our model. Yet, this can be understood in terms of the latter: Indeed, it has been stressed that the conceptual network of language is organized as a small-world network [2], and we have proposed that major semantic changes, characterized by the latency-growth pattern, would correspond to a leap from a cluster to another. It means that latency involves only one bridge, so that the set-up we explored should be enough to cover it. Growth, on the other hand, depends on the cluster size, and on the inner organization of the cluster. It thus involves a varying number of contexts, which explains why the variance of the growth would be greater in actual data, leading to a smaller Péclet number.

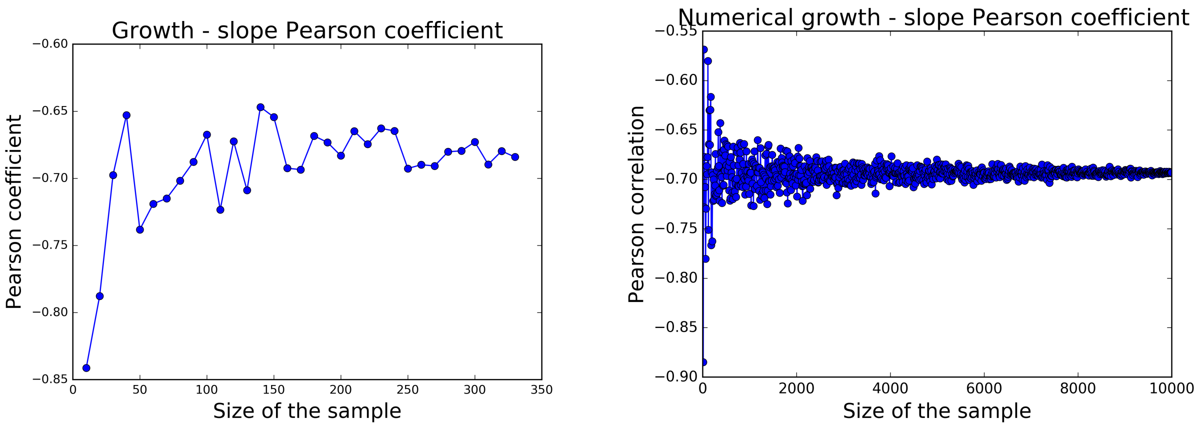

(a)

(b)

FIG. 7. Pearson coefficient for the correlation between growth time and slope obtained from (a) corpus data and (b) numerical simulations

Concerning the scale of the process, it could be tempting to compare mean latency between model and data to find the value of M (size of the memory) which would correspond to the data. However, the scale entangles both M and the size of the counting window. It also depends on the total number of involved contexts. There is thus no obvious way to compare the scales involved in the model and in the data.

2. Growth-Slope correlation and scaling law

Growth and slope are expected to be correlated. The two quantities are convincingly negatively correlated, both in corpus data (Pearson coefficient of -0.69, Fig.7a) and in our model (Pearson coefficient also equal to -0.69, Fig.7b).

It is also worthy to consider the possibility of a scaling law between these two quantities, in line with what has been evidenced for other socio-cultural changes [3], where an exponent $\alpha = -2/3$ is found between the rate h and the width w (slope and growth time, respectively). This exponent differs from the expected -1 exponent which would be expected for pure sigmoids. Our model also predicts such a scaling behavior with an exponent of $-2/3$. However, the corpus data is not characterized by any specific scaling law: The rate h and the width w are related through a trivial -1 exponent (Fig. 8):

$$\log h = -1.01 \log w + 2.10\,. \tag{21}$$

The discrepancy between the scaling behavior of corpus data and that of numerical data is yet to be explained. Once more, it could be due to the difference between the model set-up (one site competition) and the whole process of a semantic expansion (pervasion of a cluster of the semantic network), but this is purely conjectural.

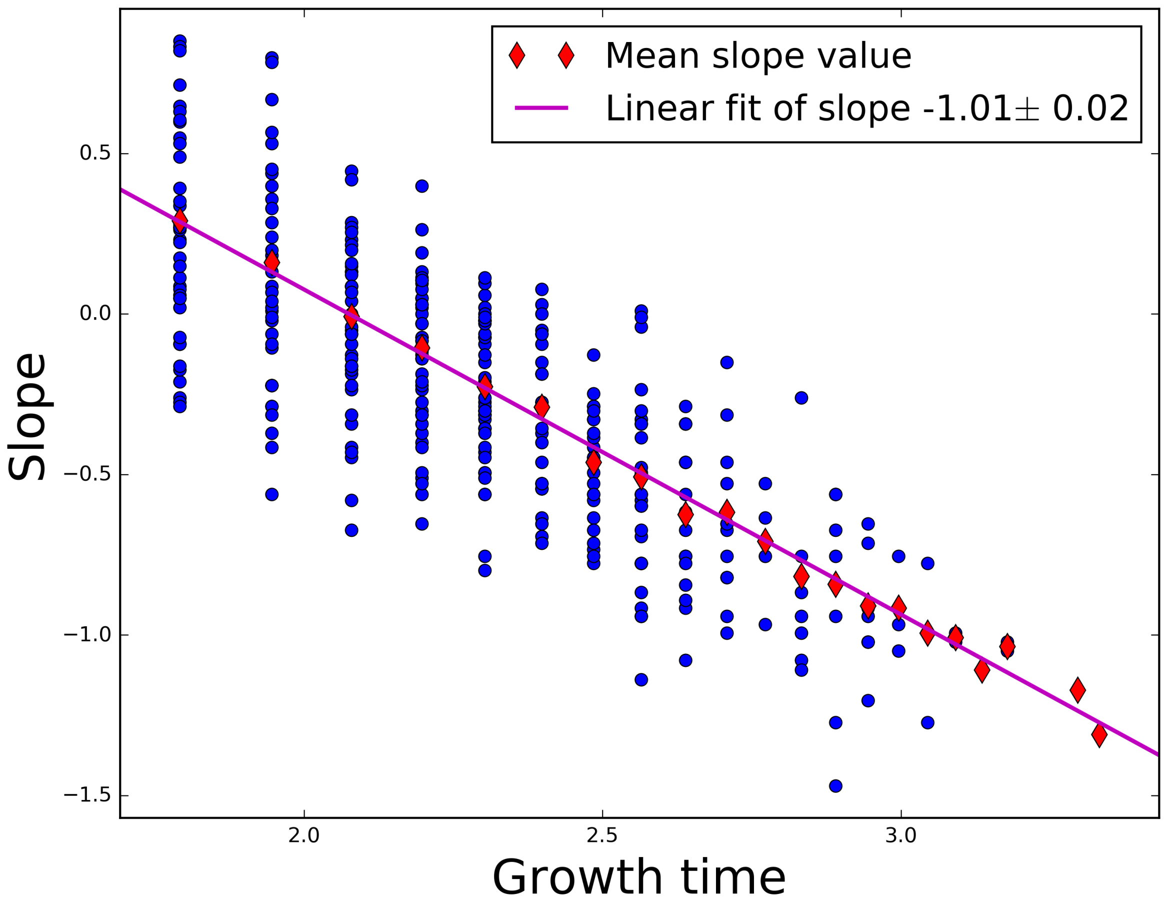

FIG. 8. Scaling law between the slope h and the growth time w. The fit is performed on the average values of the slope h for all different w. The associated r^2 is equal to 0.994.

3. Latency-Growth correlation

It may be intuitively expected for latency and growth times to be correlated: The longer the wait, the more momentum is gained. Yet, according to our model, there is no such correlation: Latency and growth times, as seen as first passage times in different parts of a Markov chain, are strictly independent quantities. However, in the empirical procedure, these two parameters become correlated, for the latency is defined as the time spent in a region comprised between $x_{t_{out}} \pm a(1 - x_{t_{out}})$, where $x_{t_{out}}$ is the frequency attained at the beginning of the growth process and a is set to 0.17 (and 0.15 for corpus data). Thus, the higher this $x_{t_{out}}$, the smaller the margin, so that a high $x_{t_{out}}$ will be correlated with a short latency, as well as a shorter growth on average. These two quantities are thus weakly positively correlated, with a Pearson coefficient of 0.20 (Fig. 9b).

If we now turn to corpus data, we find a Pearson coefficient of 0.19 (Fig.9a). The correlation between latency and growth is weak, and can be entirely imputed to the details of the empirical procedure, as we have just seen for the numerical data. It thus means that growth time and latency time are two independent quantities, so that positing a Markovian

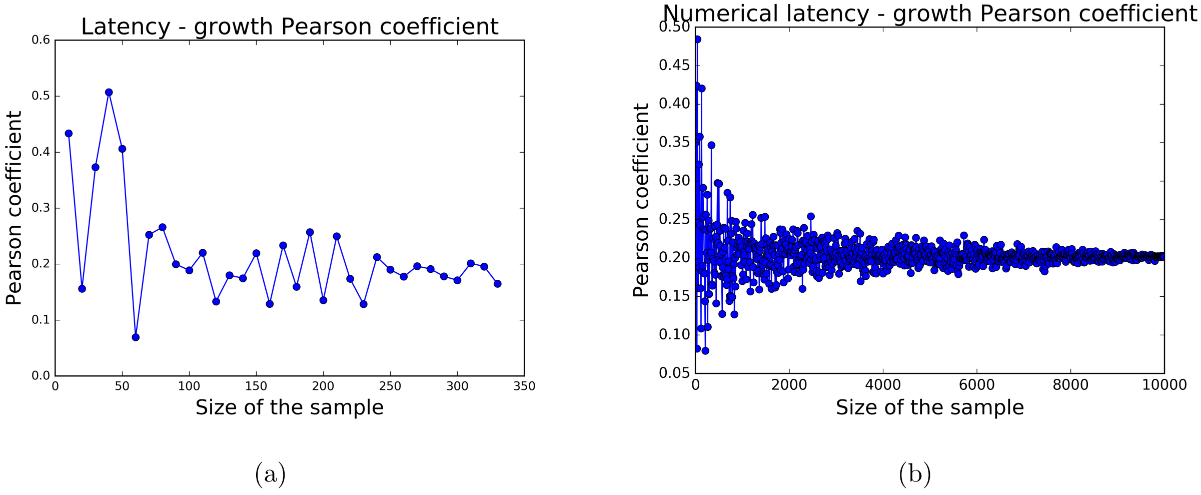

FIG. 9. Pearson coefficient for the correlation between growth time and latency time obtained from (a) corpus data and (b) numerical simulations

nature of language change is in line with findings from corpus data.

The latency and the slope are expected to be weakly negatively correlated as well, as a result from the scaling relation between the slope and the width. In the data, we find a Pearson coefficient of -0.16, to be compared with -0.23 in the model.

II. MODEL VARIANTS

A. Hearer mechanism

The model we propose in the paper describes a mechanism associated with language production: It is solely based on a speaker perspective. Yet, language change may not come only from innovation in producing language, but also in understanding it. Actually, these two aspects cannot be separated: If an innovation is possible in a speaker perspective, it must also be accessible from a hearer perspective. Be it a speaker or a hearer, a language user relies on the same cognitive entity. It seems thus necessary to consider model variants where the novelty can come from this complementary perspective, as well as from a combination of the two.

1. Hearer variant

Let us consider the same situation as for the listener model: There are two meanings, C_0 and C_1, to which are attached a pool of memories of linguistic tokens. Initially, C_0 is populated by X tokens only, while C_1 is populated by Y tokens only. Just as context C_1 is fed by the memory of C_0 when it came to express C_1, if a linguistic occurrence yields meaning C_0, it can elicit meaning C_1 as well. Occurrences of X thus have a chance to populate context C_1, so that we will note x the proportion of X tokens in C_1, just as we did in the speaker-based model. If we ascribe to the inference $C_0 \Rightarrow C_1$ a probability equal to

γ, then we can describe the dynamics as follows:

1. Either C_0 or C_1 are chosen to be expressed, with equal probabilities.
2. If C_0 has been chosen, X is produced. If C_1 has been chosen, X is produced with probability $P_0(x)$, otherwise Y is produced. $P_0(x)$ is the same function as $P_\gamma(x)$, except that γ is now set to 0 (there is no such thing as an effective frequency in this framework).
3. The produced occurrence is recorded in the chosen context. If C_0 has been chosen, an additional occurrence of the same kind as the previous one is recorded in C_1 with probability γ (C_0 has elicited the meaning C_1).
4. A past occurrence is deleted whenever needed, so as to keep both memory sizes constant.

These dynamics correspond once more to a random walk where the jump probabilities, forward and backward, respectively $R^H(x)$ and $L^H(x)$ (where H stand for 'hearer'), are given by:

$$\begin{cases} R^H(x) &= \frac{1}{2}\left[\gamma + P_0(x)\right](1-x) \\ \\ L^H(x) &= \frac{1}{2}(1-P_0(x))x \end{cases}, \tag{22}$$

to be compared with the jump probabilities in the speaker perspective (respectively $L^S(x)$ and $R^S(x)$ for the forward and backward jump probabilities):

$$\begin{cases} R^S(x) &= P_\gamma(x)(1-x) \\ \\ L^S(x) &= (1-P_\gamma(x))x \end{cases}. \tag{23}$$

These modified jump probabilities lead to a new expression for the drift velocity:

$$\dot{x} = \frac{1}{2}\left[P_0(x) - x + \gamma(1-x)\right]. \tag{24}$$

A change of variable $y = (1+\gamma)x - \gamma$ leads to the same equation as equation 4 of the paper, with a slightly different timescale accounting for the fact that two contexts are now being called:

$$\frac{2}{1+\gamma}\dot{y} = \left[P_0\left(\frac{y+\gamma}{1+\gamma}\right) - y\right]. \tag{25}$$

Indeed, $P_0\left(\frac{y+\gamma}{1+\gamma}\right)$ is exactly $P_\gamma(y)$, so that the fixed point in the hearer perspective x_c^H will be given, as a function of the fixed point x_c^S of the speaker perspective, as:

$$x_c^H = \frac{x_c^S + \gamma}{1+\gamma}, \tag{26}$$

which is higher than x_c^S. This means that, in the hearer pespective, the latency frequency will also be higher. However, it does not entail that the change will be more or less likely to happen, since what triggers the change is the fact that γ is equal to γ_c or higher, and this parameter γ_c remains the same throughout the perspective shift.

2. Combined model

We can now combine the Listener and Hearer perspectives, by taking into account the effective frequency f instead of the actual frequency x in step 2 of the dynamics outlined in the previous subsection. Then, in the above formulae, all $P_0(x)$ become $P_\gamma(x)$ (or equivalently, $P_0(f)$). The velocity is now set to:

$$\dot{x} = \frac{1}{2}\left[P_\gamma(x) - x + \gamma(1-x)\right] . \tag{27}$$

Setting $X = (x+\gamma)/(1+\gamma)$, we get:

$$2(1+\gamma)\dot{x} = P_0(X) - X + (1-X)\gamma(2+\gamma) . \tag{28}$$

We can now define a renormalized parameter $\tilde{\gamma} = \gamma(2+\gamma)$ to make this velocity similar to the one given by (24). Setting $Y = (1+\tilde{\gamma})X - \tilde{\gamma}$, we finally get:

$$2\frac{1+\gamma}{1+\tilde{\gamma}}\dot{Y} = P_{\tilde{\gamma}}(Y) - Y . \tag{29}$$

This implies that $(Y_C, \tilde{\gamma}_c) = (x_c^S, \gamma_c^S)$, so that the critical point (x_c^T, γ_c^T) in this combined perspective is equal to:

$$(x_c^T, \gamma_c^T) = \left(\frac{x_c^S + \gamma_c^S}{1+\gamma_c^S}, \sqrt{1+\gamma_c^S} - 1\right) . \tag{30}$$

In this case γ_c^T is lower than its hearer and speaker perspectives counterparts. It entails that the change would happen more easily. x_c^T is somewhere in between x_c^S and x_c^H.

3. Summary

All three variants of the model give rise to the same picture of sigmoidal growth preceded by a period of latency. The data does not allow to discriminate between either one of these three possibilities. Yet, the hypothesis that the change is driven by both hearer and speaker mechanisms is the most probable, as all language users adopt the role of hearer and speaker alternatively. An enthralling perspective of research would be to devise a quantitative criterion so as to see which of the three mechanisms best account for real language data. One could also investigate which features of language change speaker and hearer perspectives are respectively able to account for independently, and if some features need the conjunction of both to appear. Obviously, all those questions hinge upon available data and the finding of relevant observable quantities to look at.

B. Interpretations of the cognitive strength γ

In the proposed model, we make the assumption that all memory sizes are equal in the speaker perspective, and that all meanings C_i are expressed with equal probability in the hearer perspective. Here we consider the alternative that the links in the network are not weighted: They are either 1 or 0. The asymmetric structure between the two contexts C_0 and C_1 is however maintained (i.e. the graph is a directed graph and the link between sites C_0 and C_1 is 1 while the link between sites C_1 and C_0 is 0).

1. Heterogeneous memory sizes

Now let us assume different memory sizes for the two concepts, denoting by m and M the memory sizes of C_0 and C_1, respectively. Then the effective frequency of X in C_1 is given by:

$$f = \frac{N+m}{M+m} = \frac{x+m/M}{1+m/M} \tag{31}$$

By defining γ as the ratio of memories m/M, we recover the same effective frequency as before.

This means that the strength γ of the cognitive link can be interpreted as a ratio between memory sizes. If all sites were connected to each other, the occurrences expressing the contexts whose associated memory is the greatest would spread all over the network. However, not all sites lead to all others: There are pathways in the conceptual organization, which constrain possible semantic changes and allow for low-memory contexts to invade higher-memory ones.

The main difference brought forth by this interpretation is that it allows for γ's greater than one. In general, there would be no critical behavior and thus no latency, except if the conquering occurrence type comes from a very low memory context. This would suggest that, as grammaticalizations are well-characterized by the latency-growth pattern with sigmoidal increase, lexical meanings are allocated a much smaller memory than grammatical ones. However, it would also be the case within the lexicon, when a word goes from a concrete meaning to an abstract one.

It is not clear why functional and abstract meanings should be allocated a greater memory than concrete meanings. There could be for instance some advantage in making the more abstract and structural part of the conceptual realm more stable in their linguistic expression than other parts of speech, especially because they serve to constrain the processing of utterances and provide structure to the flow of speech. Were it the case, then we could understand the strong asymmetry evidenced by grammaticalization — the fact that lexical forms are recruited to express grammatical meanings overwhelmingly more frequently than the reverse. Indeed, if the links were from the stable (i.e. supported by a large memory size) to the unstable parts of the language, then all those links would be associated to a very high γ parameter, so that all parts of language would soon come to be expressed by the grammatical forms. This would right away lead to a complete communicative failure. There would thus be an obvious advantage in preventing the links from grammatical concepts to lexical ones, hence in the unidirectionality exhibited by grammaticalization.

2. Different probabilities of use

We now introduce different calling probabilities for C_0 and C_1 in the hearer perspective. Let's say that the probability to call C_0 is α. Here again γ is set to 1 (i.e. C_0 automatically entails C_1). The jump probabilities becomes thus:

$$R^H(x) = \left[\alpha + (1-\alpha)P_0(x)\right](1-x) \tag{32}$$

and:

$$L^H(x) = (1-\alpha)(1-P_0(x))x\,. \tag{33}$$

We can factorize $R^H(x)$ by $1-\alpha$. Then we recover the same computation as before, with the ratio of calling probabilities $\alpha/(1-\alpha)$ playing the role of γ. Furthermore, if we set the call probability to be proportional to memory size, then we recover the same γ as in the preceding subsection. This assumption seems natural, since greater memory sizes would help stabilizing the linguistic expressions of widely used meanings.

In such a case, the near-criticality associated to the latency-growth pattern is recovered only if the links in the conceptual network are from the seldom called contexts to the often called contexts (so as to insure low enough values of γ). This seems a natural assumption for grammaticalization phenomena, since functional meanings are much more frequently called than lexical ones. Such assumption remains of course to be carefully investigated.

These two interpretations of the cognitive link point in the same direction: In short, the links of the conceptual network would be distributed so as to prevent highly frequent forms from invading the less frequent ones, i.e., to ensure linguistic diversity. The asymmetry evidenced by grammaticalization would thus be a consequence of the fact that the highly pervasive functional forms must be kept away from the lexical, referential, more context-specific forms. This puzzling unidirectionality could thus have been selected as a cognitive structure able to guarantee a wide spectrum of possibilities in linguistic expression.

C. Sociolinguistic interpretation

We can give our model a completely different interpretation, taking a sociolinguistic view point. Instead of sites C_0 and C_1, we consider two separate communities of speakers, C_0 and C_1. Different tokens represent now different individuals, who make binary choices between either variant X or variant Y. The different community sizes, m and M, are then the analogous of the different memory sizes. The fact that C_0 influences unilaterally C_1 may be understood as the fact that community C_0 has some prestige compared to C_1, so that C_1 members listen to C_0 members while the reverse does not hold. Similarly, different call frequencies may represent different representations in society — people from prestige communities being given media visibility to the exclusion of the other communities. With this purely sociolinguistic interpretation, the model formalism thus remains exactly the same. Note that this point of view is akin to the one in [4].

In this interpretation, however, the model does not explain why the prestige community C_0 adopted X in the first place; nor does it explain the regularities in semantic change. Another point in which this interpretation weakens is the timescale. Linguistic change can be very slow, taking up to several centuries, as shown in our corpus study. Is it reasonable to presume that the social structure holds and remains the same throughout centuries? On the contrary, some aspects of conceptual structure happen to be extremely stable, as they are both deeply constitutive of a culture, e.g. through entrenched metaphors [5], and due to the generic cognitive features of the mind (expressing time relations through spatial ones [6], for instance). As it happens, metaphors prove to be very stable, even if the reasons for this stability are still unclear. The astonishing persistence of myths schemata through the ages [7] is another hint of the remarkable resilience of human cultural features.

A last remark is in order. Sociolinguistic explanation describes change as happening through two successive steps [8]: 'actuation' of the change (the seemingly sudden appearence of a new variant in the speech of an individual), and propagation of the innovation through social ties. Though Labov deemed actuation as irrelevant for the understanding of language change, numerous efforts have been devoted to make sense of it [9, 10]. Recent modeling

attemps, following Labov claim, have eluded the difficulty, positing a non-zero initial frequency of the new variant, or assuming that an influent agent is already making use of the variant exclusively [4, 11]. Latency, in particular, cannot fit within this framework.

The actuation step, on the contrary, has received much attention in Cognitive Linguisitcs and more specifically in the literature on grammaticalization. Indeed, in grammaticalization phenomena, it appears that the actuation process is tightly constrain: not all innovations are equally likely, and changes appear to follow a limited number semantic chains. Several mechanisms of actuation have thus been proposed: invited inference [12], conventionalization of an implicature [13], subjectivation [14]. They all bring forth the idea that a novel variant is always rooted in language use, so that a new form, or a new meaning, always arises out of a contigency from an existing speech practice. Actuation of the change is then an expected result of a particular cognitive organization of language.

We showed that this process of cognitive actuation is sufficient to explain the S-curve. In a sense, the cognitive interpretation is more economic, as it explains the S-curve (and the latency) by the mechanism of actuation alone, instead of positing a prerequisite actuation, and then explaining the S-curve (but not the latency) as social propagation, which is the case in the sociolinguistic framework. Occam's razor inclines therefore towards the cognitive interpretation of our model and of language change in general.

III. CORPUS DATA

A. Raw data

Raw data has been made available as a downloadable folder `full_data.zip`. To each studied linguistic form corresponds a file in this folder, named `form.csv`. This file contains a 70 rows table specifying, for each decade starting with 1321-1330, the number of occurrences of the form found in the corpus, the associated frequency, and the associated averaged frequency (over five decades, as described in Materials and Methods). Two additional files, respectively named `corpus_stats.csv` and `corpus_complet.csv`, encode all needed information on our corpus. The former is a 70 rows table listing all decades, and giving the number of occurrences associated with each (required to compute the frequency in the individual forms files). The latter is a list of all documents included in the corpus, identified by their Frantext ID. The corresponding date, the corresponding decade, and the associated number of occurrences are also specified.

B. Frantext textual database

The data we collected for the present study have been extracted from the *Frantext* database [15], one of the most extensive databases available in French, to which one has access under subscription by the ATILF-CNRS laboratory. Frantext is an ever-expanding gathering of 4,746 texts to this day (8th december 2016), updated every year. This corpus presents various literary genres (epistolary, drama, poetry, essays, scientific books), but mainly novels, almost exclusively from French literature (with a few translated works). The publication year of the texts range from 950 to 2013. The allotment of the texts between the different time periods is however far from being homogeneous, and most of them belong to

the twentieth century: Indeed, the number of texts by decade roughly follows an exponential increase (Fig. 10).

Frantext, while being much smaller than Google Ngram, provides much cleaner and more controlled results (see III C). We decided to start from the decade 1321-1330, as from this date all decades are associated with at least seven texts. In our corpus, we retained most of the texts, with a few exceptions, e.g. when the date provided by Frantext was unsatisfying (for instance, the text referred to as 6205, *Le Canarien, pièces justificatives* is dated 'between 1327 and 1470'), or when we knew that the text has been written over too long a time period, as is the case for the text *Chartes et documents de l'abbaye de Saint-Magloire* (ref 8203), whose publication year (1330) is far from covering the time span during which the document was compiled. Most interestingly, Frantext also provides the surrounding text on which a token is to be found, so that it is possible to check if the different occurrences make sense and truly correspond to the request.

Frantext is not flawless. Some parts of the scanned texts have been appended through posterior editing. This is clearly the case for the text A017, *Chroniques de Morée*, where some page notes from a contemporaneous edition of this medieval chronicle have been included, so that the request for 'dans' may return an occurrence such as 'Erreur dans la numérotation de l'édition' ('error *in* the edition numbering'). Some decades are also strongly unbalanced in the available texts. For instance, among the 2.7 million words of decade 1551-1560, more than one third of them comes from the works of a single author, Jean Calvin (references E198, B022, R849 to R852). Another bias comes from the fact that drama pieces, up to the end of the Modern Era, were making use of represented orality [16] much more than literary texts, so that many new constructions appear in them before spreading among the other texts. This would not be a problem if the proportion of dramas were more or less constant across the decades, which is not the case. This problem vanishes in more recent times, when represented orality appears also frequently in novels, while drama becomes itself

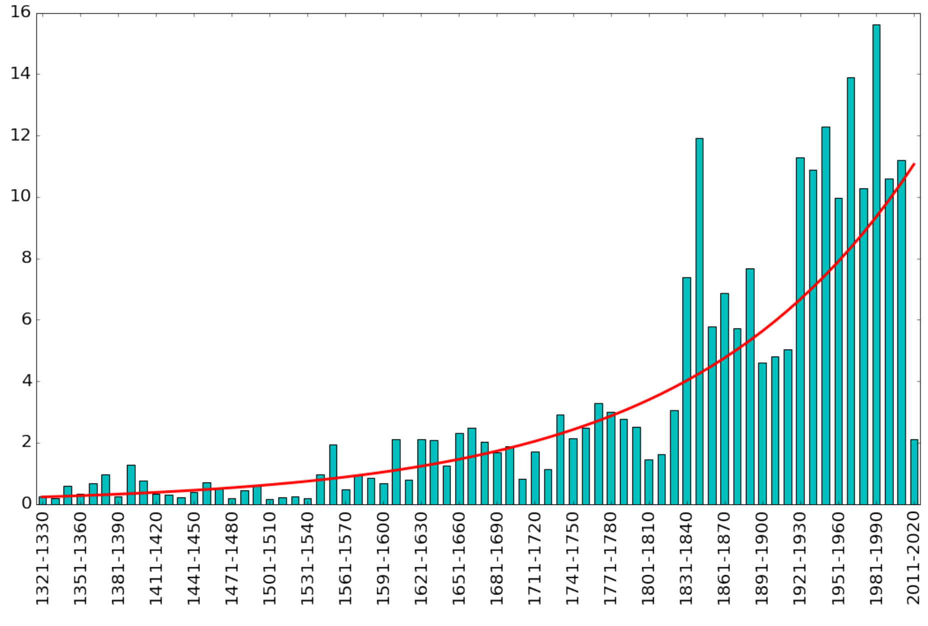

FIG. 10. Number of millions of occurrences per decade in the Frantext database. Exponential fit is shown by a red line.

more sophisticated and shifts further away from daily language.

Frantext is not only a database. It comes also with built-in text-mining algorithms which allow to submit very refined queries to the database. Such queries can make use of booleans and a given number of blank words. For instance, the query **(à|a) &q(1,2) (insçu|insu|insceu)** (**&q(1,2)** is a blank slot for any one or two words) will retrieve occurrences such as *à l'insu*, *à leur insu*, but also *à son propre insu*. This kind of flexible requests are especially relevant when one is looking for specific constructions with a filling slot, as the corresponding possibilities cannot be exhaustively predicted. We studied for instance the construction *d'une voix* + ADJ. If we cannot list all adjectives, we can rule out all the parasite occurrences with an elaborated request such as ˆ**(tous|receus) d'une voix** ˆ**(que|qui|qu'|et|ensemble|trestous|de|d'|vous|le|la|les|par|pour|dont|-|.|;|,|:)**, where ˆ and | respectively stands for the booleans 'not' and 'or'. Such a request makes it possible to capture unexpected adjectival constructs such as *toute changée*, *si peu effroyée* or *extraordinairement rauque et rouillée*, while discarding all spurious occurrences. Frantext also allows for special requests, for instance if one wishes to encompass several orthographic variations in a single query, for instance **souventes?f*** captures all possible variants of *souventesfois*, such as *souventeffoiz*, *souvente fois*, *souventez fois*, *souventefoys*, etc. This kind of elaborations prove to be all the more useful in the first stages of the evolution, where a functional construction has not yet become entrenched into an idiomatic form and can still be found in a large diversity of variants.

Once a request is submitted to the database, Frantext returns a datafile whose contents may vary according to the needs of the user. Depending on the options one chooses, the file displays, for each text, the text reference, the publication year, and the total number of occurrences of the query in that text. Next to this automatized procedure, we can also look across all individual occurrences in their context, as a sanity check. This was used frequently to help refining our queries. Unfortunately, it was impossible to ask Frantext for a file providing the statistics of the corpus itself, listing the number of occurrences per text reference. We extracted this information from an HTML page which does display this information (Corpus de travail > Visualiser). The data file provided by Frantext was then directly treated by our own algorithm to compute average frequencies for each decade.

A note on French

We acknowledge that we restricted ourselves to instances of semantic expansions in French, a choice which may appear to restrict the scope of our findings. As we argue in the main text, we believe this is not the case. In the following, we stress, 1 - the necessity to conduct the analysis on a long timescale (i.e. long enough so that we can consider the language to have changed during that period, just as contemporary French has drifted sufficiently away from Middle French (XIVth century) so that, without specific training, the latter is only partially intelligible to speakers of the former), 2 - that few corpora are as efficient as Frantext to achieve such a goal.

Given the issues addressed in this paper, it appears important to consider instances taken from a large time period (seven centuries in our case). Indeed, a frequently asked question is whether or not recent technological advances (radio, TV, the Internet) have had an influence on the way language changes. Sociologically, this influence is obvious: Languages tend to homogenize over greater geographical areas and dialects have constantly declined throughout the twentieth century. Yet, the pattern of change of an established language is something

entirely different. Our statistical survey shows that the pattern of change is the same, no matter in which century it may happen. It is furthermore consistent with recent findings establishing that the rate of change did not increase in the most recent decades [17]. It also goes along our claim that the pattern we exhibit is cognitively driven by memory retrieval and conceptual organization, two cognitive mechanisms that the most recent technological evolutions could not have significantly altered.

Alas, finding appropriate corpora covering a long time period in a given language is not obvious. As discussed in III C, Google Ngram cannot be used for texts earlier than the nineteenth centuries, since the scanning procedure does not lead to reliable digital data. For the English language, the reputed British National Corpus restricts itself to the twentieth century. The Helsinki Corpus spans a time period suited for our purposes, but the texts are too sparse (450 in total) for the corpus to be fitted for a statistical survey. The CORDE corpus, in Spanish, spans several centuries (XIIIth to XXth), and gathers an impressive amount of data as well (250 M words), but it covers different variants of Spanish (Argentinian, Colombian, Castillan, etc.) which cannot be blended together when it comes to investigate semantic expansions (note that CORDE dutifully offers to treat them apart, but then the database is not extensive enough for each of the variant separately). The querying system also suffers from serious limitations, and it is not possible to submit complex queries as is the case with Frantext. This latter database is therefore truly remarkable in many aspects and has to be considered an exception. We thus leave to further studies the case of other languages.

A last remark is in order: We deliberately do not provide any translation of the studied forms (III D), however obscure they may appear to the reader. Indeed, these forms have all undergone a semantic expansion, so that a translation would be most mistaking as it would concern only one among several meanings adopted by the form. The only satisfying way of glossing the items we studied would have been to find forms which not only have the same meaning, but have also undergone (at least roughly) the same meaning shifts, as in the case of *anyway* and *de toute façon* for the later stages of their respective semantic evolutions. Obviously, this would have been possible only for a handful of cases, and we chose to leave the items without translation.

C. Why not using Google Ngram?

Google Ngram (`https:/books.google.com/ngrams`) gathers an impressive quantity of digitalized books from about the sixteenth century. It hosts about 800,000 texts in French (about two hundred times more than Frantext). Nevertheless, it presents some major limitations which make this database inappropriate for the present study, as we discuss in this section.

Some biases of the Google Ngram database have already been stressed in the recent past [18]. However, these concerns are specifically relevant for lexical changes, most subject to socio-historical contingencies, and they do not straightforwardly apply to our aims. Functional words, unlike proper names like ‘Frodo’ or items like ‘computer’, are not that sensitive to cultural shifts. However, there are other serious limitations, more crucially relevant for our study, that we point out here. In the following, we use Google Books (`https://books.google.com/`) as a probe to the contents of Google Ngram, though the two algorithms are different (e.g. the former does not regognize ponctuation while the latter does), and the exact overlapping between the contents of the two databases is unknown.

The first concern about Google Ngram regards quality of digitalization. Texts older than the nineteenth century have been printed in fonts for which the character recognition algorithm has clearly not been optimized. For instance, the following sentence from *The royal dictionary abridged, in two parts*, by Abel Boyer, 1715: 'Parler avantageusement de quelqu'un, to speak well of one, to speak much to his advantage, to give a good character of him, to speak honourably of him.' has been transcribed as: 'Parler avantageusement e quelqu'un, 1° speak well of one, te steak much to his advant 1ge, to ive a gead characier of him, to steak h2nourably of him.' Some words, such as 'steak' and 'rince', consequently appear much more frequently than they should, as they are mistaken for 'speak' and 'Prince'. Another example of this poor scanning quality can be seen in the comparison between: 'I found that the New-modelling of this Story, would force me sometimes on the difficult Task of making the chiefest Persons speak something like their Characters, on Matter whereof I had no Ground in my Author.' and 'I faura that the Ne: -we kling of this Story, troi'i fr e ve { ctives on the di ili 7 k of making ti e li fist Perffns steak { like their Carefiers, en -i/attro sviereof. I had no Gréard in , Author.', to be found in *The History of King Lear, A Tragedy. Acted as the King's-Theatre.* by Nahum Tate, 1736. The original text is admittedly hard to decipher, yet any posterior check on the scan would immediately detect such nonsensical concatenations of characters. By comparison, every text in the Frantext database has been digitalized with great care and such blatant errors are not to be found.

The second point is the kind of available data. Google Ngram provides statistics on n-grams, which are strings of n successive items (the so-called 'grams'), with n ranging from 1 to 5. For each n-gram, it is provided, per year, the number of times it appears and in how many texts. Thus one cannot identify in which texts it appears most; nor can one have access to its context of use. The only way to probe the contents of Google Ngram is through Google Books (which we used here for all the discussed examples), yet it seems impossible to know the exact overlap between the two databases. This data structure based on n-grams is furthermore limiting when it comes to slot constructions. For instance, the French construction 'à X reprises', with X being a quantity, can hardly be tracked using Google Ngram, as it corresponds to far too many n-grams, which would need to be listed one by one: 'à deux reprises', 'à deux ou trois reprises', 'à plusieurs reprises', 'à de nombreuses reprises', etc. This search is made all the more difficult by the fact that 'à' did not always take an accent in older texts. In contrast, with Frantext, as we have seen in III B, one can work out an elaborate request using booleans and blank words to capture the diverse uses of this construction and overcome the orthographic difficulties.

The third and final point we want to stress here is the choice of texts and their dating. In Frantext, a text may appear in several editions, as is the case for *Le Cid*, by Pierre Corneille, which appear thrice in the database, associated to the years 1637, 1637 and 1682. These dates usually correspond to the first edition of a book, rather than to the edition which is actually digitalized (such information being also provided). Google Ngram displays about thirty versions of *Le Cid*, with publication ranging from 1775 to 2013, some of them being ascribed to Jean Racine (as they are found in several editions of a book called *Oeuvres de J. Racine et de P. et T. Corneille*). The case of *Le Cid* is, in Frantext, quite an exception, while in Google Ngram, most famous classical novels from past centuries are found in a dozen versions at least.

The contents of the database is problematic as well. As highlighted in [18], Google Ngram over-represent academic literature. This also tends to bias the data. For instance, among the fourteen results of the request 'par ma barbe' on the French Google Books subdatabase,

for the years 1950-2000, only three of them are relevant, two being modern translations of older texts (Don Quixote and a nineteenth century German play by Töpffer). The third one comes from an anthology of French folktales. All other occurrences are academic quotes and glosses of past works, or reprints of such works. In such a case, it means than only one fifth of the occurrences would be reliable as a reflect of language use in this time period (two of them being borderline cases). Frantext, on the other hand, has two occurrences of 'par ma barbe', one of them from the song lyrics of singer Georges Brassens, the other from a 1988 translation of a Shakespeare play (and so more debatable). There is thus almost as many relevant occurrences in Frantext and Google Ngram (two versus three), while none in Frantext are completely irrelevant.

This being said, Google Ngram is a formidable tool, which can lead to interesting insights and be of great use. It is not, however, fitted for the work that we performed, where we need an accuracy and a reliability that this database is unable to provide.

D. Studied forms

Making use of the study of Frantext database and its retrieving tools, we looked at the frequency of use of about 400 hundred semantic expansions in the functional realm (with the exception of *liberté* and some lexical constructions such as *court terme*, which we have shown to suggest the further generality of the pattern). We selected these forms according to several criteria: They must have undergone at least one semantic expansion towards a functional use during the time period under consideration; they must be easily distinguished from compositional uses (e.g. *entre deux*, in the meaning of 'in between', can be confused with occurrences of literal meaning 'between two'). The set of chosen forms is far from exhausting the pool of possible examples.

On the table below, we provide the full list of studied forms. For each of those, we display:

- the length (in decades) of the latency part;
- the length (in decades) of the growth part;
- the slope of the logit transform of the growth part;
- the r^2 parameter associated to the linear fit of this logit transform;
- the χ^2 of the sigmoidal fit of the data (including the boundaries x_{min} and x_{max});
- the associated Cramér's V (which is the square root of the ratio between the χ^2 and the width, or the square root of the mean χ^2 per data point; the smaller the Cramér's V, the better the fit)
- the result of the consistency check (either ✓if successful or $\times$ if failed), as described in section I A 3;
- the total number of occurrences of the form in our corpus.

Some forms are listed several times; it corresponds to the case where a form underwent several semantic expansion processes, each associated with the latency-growth pattern. 'BUG' corresponds to a flaw of Frantext, sometimes unable to build up the output file of the query. This bug cannot be overriden through a manual manoeuvre, for it is caused by a

faulty encoding of some parts of the texts. The data thus exist, but could not be retrieved. An upper-case 'NO' indicates that no such pattern has been found in the time-evolution of the frequency of that form. The fact that a form does not follow an S-curve during its semantic expansion may spread doubt on the genericity of this pattern. In many cases however, the pattern was rejected because the data was too spurious, but its overall behavior would not be incompatible with an S-curve.

It is nonetheless interesting to note that the robustness of the pattern does not depend excessively on the scarcity of data. Indeed, instances associated to a very low number of occurrences can lead to a very clean pattern (e.g. *à plus d'un titre*, whose growth lasts for 8 decades in total, scores as low as 59 occurrences, and still brings out a remarkable r^2 of 0.995). What seems to be crucial is thus not the question of how much data we can get, but of whether or not the change is isolated. Indeed, some changes are not independent from one another. Many constructions beginning with the preposition *par*, for instance, follow their own course of evolution, while the meaning of *par* itself also expands. Several constructions can also compete for the same paradigm (e.g. *il me semble*, *je pense*, *je suppose*). Their individual frequency pattern not following an S-curve of growth may thus be seen as resulting from interferences between the different semantic expansion processes. In these cases, only the refinement of linguistic queries can lead to better results. It thus confirms, once again, the necessity to rely on a clean and easily manipulable database rather than on giant databases where the sheer amount of data is of no help.

LIST OF FORMS

Form	Lat.	Growth	Slope	r^2	χ^2	C.'s V	Check	# occ.
à base de	7	10	0.57	0.994	0.1097	0.1047	✓	607
à bien des égards (i)	0	8	0.79	0.983	0.0602	0.0867	✓	147
à bien des égards (ii)	2	7	1.27	0.984	0.0567	0.0900	✓	147
à bord de	NO	NO	NO	NO	NO	NO	NO	1728
acabit	0	7	1.20	0.992	0.0345	0.0702	✓	148
à cause de	NO	NO	NO	NO	NO	NO	NO	24840
à cause que	NO	NO	NO	NO	NO	NO	NO	2516
à ce moment (i)	0	10	0.61	0.989	0.0777	0.0881	✓	8861
à ce moment (ii)	7	7	0.69	0.992	0.0710	0.1007	×	8861
à ce propos (i)	2	7	2.22	0.988	0.0004	0.0076	✓	1711
à ce propos (ii)	3	8	0.88	0.983	0.0623	0.0882	✓	1711
à ce sujet	10	7	1.95	0.984	0.0217	0.0557	✓	4001
à cet égard	0	8	1.56	0.992	0.1118	0.1182	✓	4974
à cet instant	2	13	0.55	0.982	0.1123	0.0929	✓	1198
à condition de	5	9	0.79	0.991	0.0427	0.0689	✓	1151
à condition que (i)	11	6	1.19	0.997	0.0470	0.0885	✓	1653
à condition que (ii)	6	8	0.83	0.971	0.0909	0.1066	✓	1653
à contre-courant	0	16	0.59	0.971	0.0797	0.0706	✓	171
à cté de	22	14	0.57	0.965	0.0603	0.0656	✓	18065

LIST OF FORMS

Form	Lat.	Growth	Slope	r^2	χ^2	C.'s V	Check	# occ.
à coup sûr (i)	0	14	0.63	0.971	0.0916	0.0809	✓	2546
à coup sûr (ii)	7	7	1.70	0.996	0.0088	0.0355	✓	2546
à court terme	13	7	2.19	0.997	0.0072	0.0321	✓	751
à couvert	NO	NO	NO	NO	NO	NO	NO	1144
actuellement	9	24	0.35	0.977	0.1300	0.0736	×	6618
à découvert	1	7	1.33	0.981	0.0390	0.0746	✓	930
à défaut de	NO	NO	NO	NO	NO	NO	NO	1725
afin de	4	6	0.81	0.995	0.1155	0.1387	×	21833
afin que	BUG	BUG	BUG	BUG	BUG	BUG	BUG	19850
à fond de	0	6	1.31	0.994	0.0302	0.0709	✓	486
à fond de train	BUG	BUG	BUG	BUG	BUG	BUG	BUG	180
à force	NO	NO	NO	NO	NO	NO	NO	294
à force de	NO	NO	NO	NO	NO	NO	NO	8178
à grand renfort de	NO	NO	NO	NO	NO	NO	NO	230
ainsi donc	NO	NO	NO	NO	NO	NO	NO	1247
à la base	NO	NO	NO	NO	NO	NO	NO	574
à l'accoutumée	0	8	0.99	0.988	0.0327	0.0639	✓	196
à l'aide de	13	13	0.42	0.966	0.1952	0.1225	×	5247
à la limite	7	11	0.76	0.983	0.0448	0.0638	✓	603
à la lisière de	5	13	0.57	0.976	0.0638	0.0701	✓	527
à la longue	9	7	0.57	0.987	0.2092	0.1729	✓	1245
à la lumière de	2	9	0.87	0.975	0.0823	0.0956	✓	1141
à la mesure de (i)	0	7	0.75	0.994	0.0925	0.1150	✓	819
à la mesure de (ii)	24	9	1.14	0.988	0.0376	0.0646	✓	819
à la place	22	27	0.31	0.983	0.0918	0.0583	✓	5638
à la rigueur	9	8	1.14	0.983	0.0697	0.0933	✓	1717
à l'écart	NO	NO	NO	NO	NO	NO	NO	2517
à l'écart de	24	19	0.30	0.970	0.1545	0.0902	✓	854
à l'égard de (i)	4	13	1.01	0.968	0.1068	0.0906	×	13395
à l'égard de (ii)	2	7	0.98	0.978	0.0658	0.0970	✓	13396
à l'encontre de	10	18	0.39	0.977	0.0946	0.0725	✓	1272
à l'envi	0	9	0.88	0.991	0.0456	0.0712	✓	817
à l'exception de	1	16	0.47	0.968	0.1093	0.0827	✓	1883
à l'heure actuelle	0	11	0.95	0.981	0.0698	0.0797	✓	858
à l'heure dite	3	9	0.83	0.969	0.0587	0.0808	✓	234
à l'heure où	10	11	0.58	0.963	0.1015	0.0961	✓	1779

LIST OF FORMS

Form	Lat.	Growth	Slope	r^2	χ^2	C.'s V	Check	# occ.
à l'improviste	4	10	0.65	0.996	0.0549	0.0741	✓	1024
à l'instant	0	6	1.02	0.993	0.1038	0.1315	✓	1550
à l'instar de	7	19	0.36	0.969	0.1502	0.0889	✓	663
à l'insu	0	22	0.36	0.982	0.1347	0.0782	✓	2776
à l'inverse	8	10	1.06	0.988	0.023	0.0476	✓	764
à l'occasion de	6	8	1.52	0.983	0.0469	0.0766	✓	2032
à l'orée de	5	7	1.09	0.979	0.0793	0.1064	✓	311
alors que (i)	3	7	1.01	0.983	0.0474	0.0823	✓	28016
alors que (ii)	4	13	0.50	0.983	0.0700	0.0734	×	28016
à mesure de	4	8	0.71	0.990	0.1122	0.1184	×	774
à mesure que (i)	10	10	0.80	0.967	0.0850	0.0922	✓	10183
à mesure que (ii)	1	11	0.50	0.965	0.1214	0.1051	✓	10183
à moins que	0	13	0.96	0.964	0.0959	0.0859	✓	5924
à mon avis	NO	NO	NO	NO	NO	NO	NO	1989
à nouveau	7	14	0.51	0.977	0.0801	0.0756	×	6039
à outrance	NO	NO	NO	NO	NO	NO	NO	552
à part	0	28	0.27	0.986	0.1034	0.0608	✓	12506
à part entière	0	8	1.33	0.983	0.0467	0.0764	✓	180
à partir de	13	12	0.56	0.965	0.1212	0.1005	✓	10996
à peine (i)	0	6	1.73	0.994	0.0175	0.0540	✓	40230
à peu de chose près	0	7	0.94	0.987	0.0693	0.0995	✓	320
à plus d'un titre	1	7	1.02	0.995	0.0524	0.0865	✓	59
à plusieurs reprises (i)	0	19	0.36	0.967	0.1261	0.0815	✓	3873
à plusieurs reprises (ii)	9	7	1.22	0.994	0.0315	0.0671	✓	3873
après ce	NO	NO	NO	NO	NO	NO	NO	101
après que	6	6	2.34	0.997	0.0078	0.0361	✓	8487
après quoi	10	16	0.53	0.982	0.0742	0.0681	✓	3468
après tout	NO	NO	NO	NO	NO	NO	NO	7741
a priori	3	9	1.21	0.985	0.0641	0.0844	✓	1565
à propos	NO	NO	NO	NO	NO	NO	NO	1255
à propos de	1	19	0.39	0.972	0.0848	0.0668	✓	9414
à proprement parler	10	11	0.49	0.965	0.1065	0.0984	✓	1204
à rebours (i)	2	6	1.09	0.994	0.0627	0.1022	✓	640
à rebours (ii)	6	12	0.66	0.979	0.0767	0.0799	✓	640
à qui mieux mieux	NO	NO	NO	NO	NO	NO	NO	247
à sa guise	0	6	1.40	0.992	0.0503	0.0916	✓	1079

LIST OF FORMS

Form	Lat.	Growth	Slope	r^2	χ^2	C.'s V	Check	# occ.
à son terme	1	11	0.75	0.991	0.0518	0.0686	✓	359
à tel point que (i)	0	7	0.75	0.996	0.1262	0.1343	✓	555
à tel point que (ii)	4	9	0.73	0.975	0.1025	0.1067	✓	555
à terme	NO	NO	NO	NO	NO	NO	NO	470
à titre de	5	14	0.47	0.964	0.1234	0.0939	✓	1481
à tous égards	0	6	1.91	0.998	0.0138	0.0480	✓	556
à tout à l'heure	0	10	0.93	0.983	0.0655	0.0809	✓	280
à tout instant	5	11	0.94	0.969	0.0846	0.0877	✓	903
à tout moment	0	17	0.42	0.968	0.1608	0.0973	×	2262
à tout prendre	NO	NO	NO	NO	NO	NO	NO	480
au bord de	NO	NO	NO	NO	NO	NO	NO	11850
au bout de	NO	NO	NO	NO	NO	NO	NO	23173
au bout du compte	NO	NO	NO	NO	NO	NO	NO	469
au contraire	3	9	0.93	0.978	0.0606	0.0821	✓	29571
au contraire de (i)	0	8	1.09	0.977	0.0475	0.0771	✓	1429
au contraire de (ii)	1	8	1.14	0.989	0.0341	0.0653	✓	1429
aucunefois	NO	NO	NO	NO	NO	NO	NO	1248
au demeurant	0	12	0.68	0.983	0.0685	0.0756	✓	1344
au dépourvu	NO	NO	NO	NO	NO	NO	NO	402
au détriment de	NO	NO	NO	NO	NO	NO	NO	798
au dernier moment	NO	NO	NO	NO	NO	NO	NO	1370
au final	NO	NO	NO	NO	NO	NO	NO	38
au fur et à mesure	6	12	0.72	0.987	0.0340	0.0532	×	1908
au jour d'aujourd'hui	NO	NO	NO	NO	NO	NO	NO	87
au même moment	5	7	0.73	0.979	0.1091	0.1248	✓	1437
au moment o	6	19	0.49	0.984	0.0403	0.0461	✓	12729
à un moment donné	1	12	0.48	0.980	0.1249	0.1020	✓	659
au passage	0	7	1.43	0.990	0.0492	0.0838	✓	1754
au pire (i)	0	12	0.46	0.965	0.1424	0.1089	✓	401
au pire (ii)	0	6	1.63	0.994	0.0315	0.0725	✓	401
au reste	0	7	1.39	0.987	0.0350	0.0707	✓	4375
au sujet de	1	12	0.75	0.981	0.0565	0.0686	✓	4945
au terme de (i)	7	12	0.47	0.971	0.1214	0.1006	✓	1492
au terme de (ii)	1	11	0.86	0.967	0.0984	0.0946	✓	1492
aux trousses	NO	NO	NO	NO	NO	NO	NO	419
avant tout	27	10	0.91	0.986	0.0565	0.0752	✓	5342

LIST OF FORMS

Form	Lat.	Growth	Slope	r^2	χ^2	C.'s V	Check	# occ.
avec force	NO	NO	NO	NO	NO	NO	NO	324
bah	7	11	1.03	0.964	0.0579	0.0726	✓	2681
bien entendu (i)	5	10	0.76	0.985	0.0555	0.0745	✓	4476
bien entendu (ii)	2	19	0.40	0.979	0.1410	0.0861	×	4476
bien sûr	9	9	0.92	0.968	0.0839	0.0966	✓	7997
bref	12	7	1.07	0.993	0.0353	0.0710	✓	5536
brusquement	11	9	1.49	0.979	0.1103	0.1107	×	1783
carrément (i)	0	10	1.02	0.964	0.0640	0.0800	✓	1207
carrément (ii)	1	7	1.55	0.982	0.0433	0.0786	✓	1207
ce faisant (i)	0	6	1.88	0.992	0.0180	0.0548	✓	781
ce faisant (ii)	19	8	0.64	0.994	0.0918	0.1071	✓	781
ce par quoi	0	10	0.61	0.984	0.0707	0.0841	✓	163
c'est alors que	5	11	0.69	0.967	0.0732	0.0816	✓	3223
c'est pour le coup que	0	6	0.77	0.990	0.1309	0.1477	✓	64
c'est pourquoi (i)	0	13	0.56	0.984	0.0380	0.0541	×	10994
c'est pourquoi (ii)	6	15	0.59	0.968	0.1755	0.1082	×	10994
chemin faisant	0	13	0.40	0.965	0.1220	0.0969	✓	641
complètement	NO	NO	NO	NO	NO	NO	NO	11560
compte tenu de	0	8	1.26	0.985	0.0291	0.0603	✓	928
concernant	9	10	1.10	0.984	0.0615	0.0784	×	3477
considérant que	NO	NO	NO	NO	NO	NO	NO	191
contre mon attente	NO	NO	NO	NO	NO	NO	NO	102
contre toute attente (i)	0	6	0.84	0.991	0.1103	0.1356	✓	167
contre toute attente (ii)	8	8	0.95	0.971	0.0979	0.1106	✓	167
d'abord et avant tout	NO	NO	NO	NO	NO	NO	NO	62
d'année en année	NO	NO	NO	NO	NO	NO	NO	505
dans ce cas	0	18	0.57	0.974	0.1264	0.0838	×	4289
dans la mesure de	6	12	0.49	0.983	0.0723	0.0776	✓	480
dans la mesure du possible	0	12	0.64	0.980	0.0465	0.0622	✓	188
dans la mesure o	0	11	0.91	0.965	0.1322	0.1096	×	2753
dans le cadre de	11	8	1.16	0.971	0.0330	0.0642	✓	1145
dans le mme temps (i)	0	9	1.02	0.986	0.0333	0.0608	✓	1217
dans le mme temps (ii)	3	7	0.90	0.983	0.1137	0.1274	✓	1217
dans l'ensemble (i)	0	9	0.74	0.967	0.0816	0.0952	✓	1809
dans l'ensemble (ii)	10	9	0.93	0.969	0.0661	0.0857	✓	1809
dans l'immédiat	10	9	1.10	0.984	0.0303	0.0580	✓	329

LIST OF FORMS

Form	Lat.	Growth	Slope	r^2	χ^2	C.'s V	Check	# occ.
dans quelque temps	0	6	0.76	0.991	0.1579	0.1622	✓	234
dans son ensemble	1	9	0.67	0.979	0.0725	0.0898	✓	835
dans un autre temps	NO	NO	NO	NO	NO	NO	NO	143
dans un cas comme dans l'autre	1	8	1.04	0.983	0.0373	0.0683	✓	111
dans une large mesure	5	8	0.66	0.994	0.0870	0.1043	✓	381
dans un instant	1	11	0.67	0.969	0.0690	0.0792	✓	661
dans un moment	0	15	0.47	0.984	0.0673	0.0670	✓	1473
dans un premier temps	NO	NO	NO	NO	NO	NO	NO	229
dans tous les cas	5	14	0.71	0.978	0.1111	0.0891	✓	1609
d'autant plus	1	10	0.74	0.976	0.0721	0.0849	✓	11584
d'autant plus que	4	7	1.57	0.979	0.0446	0.0798	✓	3339
d'autre part (i)	0	7	0.99	0.988	0.0697	0.0998	✓	11012
d'autre part (ii)	12	12	0.64	0.982	0.0730	0.0780	×	11012
de ce cté	NO	NO	NO	NO	NO	NO	NO	3665
décidément	0	15	0.44	0.976	0.0931	0.0788	×	4795
de ce fait	2	9	0.69	0.974	0.1142	0.1126	×	628
de façon que	NO	NO	NO	NO	NO	NO	NO	1473
de fait	0	9	0.97	0.978	0.0569	0.0795	✓	5018
de jour en jour	NO	NO	NO	NO	NO	NO	NO	2217
de la part de	NO	NO	NO	NO	NO	NO	NO	16400
de la sorte	8	13	0.61	0.965	0.0855	0.0811	✓	3752
de l'aveu de	0	17	0.34	0.986	0.1072	0.0794	✓	196
de l'avis de	NO	NO	NO	NO	NO	NO	NO	146
de loin	16	10	0.73	0.994	0.0314	0.0560	✓	1262
de loin en loin	0	20	0.35	0.975	0.1541	0.0878	✓	1348
de long en large	3	9	0.76	0.983	0.0550	0.0782	✓	734
d'emblée	3	10	0.72	0.986	0.0465	0.0682	✓	1451
de mèche	NO	NO	NO	NO	NO	NO	NO	98
de mieux en mieux	0	6	1.32	0.996	0.0519	0.0930	✓	445
de moins en moins	6	21	0.28	0.980	0.1005	0.0692	✓	1536
de mon cté	0	14	0.71	0.981	0.0836	0.0773	×	8788
de mon fait	NO	NO	NO	NO	NO	NO	NO	467
de nulle part	24	13	0.32	0.978	0.1708	0.1146	✓	289
de pair	12	7	1.04	0.983	0.0575	0.0906	✓	578
de place en place	15	9	0.89	0.974	0.0463	0.0717	✓	376
de point en point	0	6	0.75	0.988	0.1511	0.1587	✓	247

LIST OF FORMS

Form	Lat.	Growth	Slope	r^2	χ^2	C.'s V	Check	# occ.
de part en part (i)	0	7	1.49	0.995	0.0218	0.0558	✓	498
de part en part (ii)	7	6	1.42	0.989	0.0386	0.0802	✓	498
de part et d'autre	NO	NO	NO	NO	NO	NO	NO	2505
de plus en plus	0	6	1.08	0.999	0.0587	0.0989	✓	18226
de près ou de loin	NO	NO	NO	NO	NO	NO	NO	221
de proche en proche	3	9	1.00	0.987	0.0273	0.0551	✓	702
de quelque part	NO	NO	NO	NO	NO	NO	NO	166
des fois	5	12	0.75	0.968	0.0770	0.0801	✓	1423
des fois que	0	10	0.75	0.977	0.0673	0.0820	✓	182
dès l'instant (i)	9	8	0.51	0.976	0.1873	0.1530	✓	769
dès l'instant (ii)	1	8	0.96	0.988	0.0622	0.0882	✓	769
dès lors que	NO	NO	NO	NO	NO	NO	NO	994
de sorte que	6	6	0.85	0.997	0.1307	0.1476	✓	11320
de surcroît	36	11	1.08	0.918	0.0811	0.0859	✓	720
de temps à autre	15	15	0.52	0.969	0.1061	0.0841	✓	3547
de temps en temps	1	14	0.40	0.977	0.1176	0.0917	✓	8916
de toute façon	35	11	0.76	0.996	0.0224	0.0451	✓	3595
de toute manière	25	9	0.66	0.973	0.0858	0.0976	✓	727
de toutes façons	4	6	2.04	0.989	0.0306	0.0714	✓	715
de toutes parts	13	10	0.81	0.972	0.0780	0.0883	✓	4792
d'heure en heure	NO	NO	NO	NO	NO	NO	NO	573
d'ici là	16	9	0.60	0.985	0.1554	0.1314	×	904
dorénavant	NO	NO	NO	NO	NO	NO	NO	256
d'outre en outre	NO	NO	NO	NO	NO	NO	NO	47
du fait de	24	8	0.73	0.986	0.0702	0.0937	✓	1423
du mme coup	14	17	0.47	0.991	0.0506	0.0546	✓	1502
du moment que	2	8	0.84	0.979	0.0646	0.0899	✓	1765
d'une manière ou d'une autre	NO	NO	NO	NO	NO	NO	NO	320
d'une part (i)	0	8	1.13	0.985	0.0354	0.0665	✓	5671
d'une part (ii)	3	7	0.80	0.982	0.0844	0.1098	×	5671
d'une voix claire (i)	6	10	0.66	0.993	0.0542	0.0736	✓	13511
d'une voix claire (ii)	4	9	0.71	0.985	0.0594	0.0812	✓	13511
du pareil au mme	NO	NO	NO	NO	NO	NO	NO	92
du point de vue de	7	8	0.80	0.995	0.0485	0.0779	✓	899
du reste	1	6	1.82	0.994	0.0178	0.0545	✓	5510
en attendant	NO	NO	NO	NO	NO	NO	NO	3351

LIST OF FORMS

Form	Lat.	Growth	Slope	r^2	χ^2	C.'s V	Check	# occ.
en attendant de	4	8	1.09	0.979	0.0641	0.0895	✓	510
en attendant que	NO	NO	NO	NO	NO	NO	NO	2270
en bordure de	0	11	0.83	0.983	0.0403	0.0605	✓	434
en bref	1	6	1.06	0.996	0.0724	0.1098	✓	339
en ce moment (i)	5	8	1.09	0.985	0.0624	0.0883	✓	12751
en ce moment (ii)	1	11	0.53	0.970	0.1012	0.0959	×	12751
en ce que	0	8	1.24	0.972	0.0544	0.0825	✓	3971
en ce qui concerne	28	14	0.47	0.973	0.1243	0.0942	×	3950
en ce qui me concerne	0	13	0.57	0.970	0.0922	0.0842	✓	682
en considération de	0	10	0.74	0.967	0.0960	0.0980	✓	409
en cours de	0	14	0.54	0.984	0.1289	0.0960	×	1110
en cours de route	0	8	0.91	0.986	0.0595	0.0862	✓	301
en d'autres termes	0	10	0.70	0.971	0.0615	0.0784	✓	1228
en définitive	NO	NO	NO	NO	NO	NO	NO	1538
en dépit de	NO	NO	NO	NO	NO	NO	NO	4016
en face de	18	17	0.39	0.982	0.1044	0.0784	×	10956
en façon que	NO	NO	NO	NO	NO	NO	NO	48
en fait (i)	0	6	0.91	0.990	0.1372	0.1512	✓	8871
en fait (ii)	44	12	0.61	0.968	0.0726	0.0778	×	8871
en fin de compte	27	14	0.43	0.972	0.0955	0.0826	✓	1417
en gros	23	10	0.86	0.975	0.0461	0.0679	✓	320
en guise de	7	10	0.93	0.982	0.0390	0.0624	✓	1598
en instance de	1	9	0.80	0.967	0.1036	0.1073	✓	77
en l'occurrence	0	11	0.58	0.993	0.0476	0.0658	✓	525
en long et en large	NO	NO	NO	NO	NO	NO	NO	108
en mme temps	3	10	0.88	0.996	0.0242	0.0492	✓	18370
en mme temps que	0	18	0.47	0.968	0.1421	0.0889	✓	8241
en mesure de	NO	NO	NO	NO	NO	NO	NO	1470
en particulier (i)	2	6	1.26	0.993	0.0352	0.0766	✓	8949
en particulier (ii)	17	15	0.39	0.984	0.1001	0.0817	✓	8949
en particulier (iii)	4	7	0.80	0.987	0.0853	0.1104	×	8949
en partie	NO	NO	NO	NO	NO	NO	NO	5645
en passe de	NO	NO	NO	NO	NO	NO	NO	46
en plein	NO	NO	NO	NO	NO	NO	NO	183
en plein qqch	31	11	0.94	0.968	0.0720	0.0809	×	15939
en quelque sorte	NO	NO	NO	NO	NO	NO	NO	6422

LIST OF FORMS

Form	Lat.	Growth	Slope	r^2	χ^2	C.'s V	Check	# occ.
en sorte que	NO	NO	NO	NO	NO	NO	NO	4786
en suspens	BUG	BUG	BUG	BUG	BUG	BUG	BUG	961
en tant que tel	8	6	0.91	0.997	0.0836	0.1180	✓	314
entre autres	NO	NO	NO	NO	NO	NO	NO	4402
en vérité (i)	0	6	0.93	0.988	0.1352	0.1501	✓	8194
en vérité (ii)	5	8	0.65	0.971	0.1104	0.1175	✓	8194
en voie de	0	24	0.36	0.970	0.1239	0.0719	✓	1027
en vue de (i)	5	6	1.31	0.995	0.0348	0.0762	✓	3625
en vue de (ii)	11	14	0.34	0.964	0.1586	0.1064	×	3625
époque	7	14	0.75	0.993	0.0340	0.0493	×	32290
essentiellement	3	7	0.80	0.976	0.0892	0.1129	×	5471
étant donné que	2	13	0.62	0.984	0.0474	0.0604	✓	341
et après	NO	NO	NO	NO	NO	NO	NO	7562
excepté	5	7	0.66	0.990	0.1250	0.1336	✓	5042
faute de (i)	5	7	1.76	0.990	0.0133	0.0436	✓	6725
faute de (ii)	7	12	0.78	0.978	0.0642	0.0731	✓	6725
faute de quoi	NO	NO	NO	NO	NO	NO	NO	262
force est de	NO	NO	NO	NO	NO	NO	NO	84
fors	BUG	BUG	BUG	BUG	BUG	BUG	BUG	4451
graduellement	NO	NO	NO	NO	NO	NO	NO	827
hormis	4	11	0.83	0.964	0.0934	0.0921	✓	1464
il me semble	NO	NO	NO	NO	NO	NO	NO	1822
il s'agit de	3	17	0.33	0.978	0.1358	0.0894	✓	11558
il y a moyen	8	8	1.31	0.979	0.0307	0.0619	✓	1295
j'ai l'impression	0	9	0.57	0.983	0.1066	0.1088	✓	74
ja soit ce que	NO	NO	NO	NO	NO	NO	NO	268
je pense	5	6	1.48	0.989	0.0258	0.0656	✓	4033
je suppose	0	8	1.00	0.995	0.0258	0.0568	✓	1110
j'imagine	NO	NO	NO	NO	NO	NO	NO	824
jusque là	NO	NO	NO	NO	NO	NO	NO	6908
juste un	26	11	0.59	0.973	0.1148	0.1022	×	1366
l'autre jour	NO	NO	NO	NO	NO	NO	NO	4438
lendemain	NO	NO	NO	NO	NO	NO	NO	28780
le temps de	20	13	0.46	0.972	0.1007	0.0880	✓	1195
liberté	2	9	0.87	0.990	0.0500	0.0745	✓	46705
l'un dans l'autre	NO	NO	NO	NO	NO	NO	NO	69

LIST OF FORMS

Form	Lat.	Growth	Slope	r^2	χ^2	C.'s V	Check	# occ.
l'un après l'autre	NO	NO	NO	NO	NO	NO	NO	2010
m'est avis	NO	NO	NO	NO	NO	NO	NO	797
nettement	0	10	0.45	0.972	0.1682	0.1297	✓	6109
nommément	NO	NO	NO	NO	NO	NO	NO	453
non pas tant	3	6	1.25	1.000	0.0312	0.0721	✓	855
non seulement	11	18	0.57	0.966	0.1195	0.0815	✓	22599
non pas seulement	2	10	0.94	0.987	0.1166	0.1080	✓	1605
notamment	28	9	0.52	0.976	0.1690	0.1370	×	7508
nulle part	5	12	0.57	0.980	0.0679	0.0752	✓	5006
or donc	NO	NO	NO	NO	NO	NO	NO	237
ouille	0	10	0.98	0.977	0.0399	0.0632	✓	106
outre mesure	3	11	0.52	0.970	0.1227	0.1056	✓	664
par à-coups	0	13	0.46	0.974	0.1224	0.0970	✓	212
par ailleurs	27	13	0.99	0.971	0.0602	0.0680	×	2676
par amour	5	8	0.87	0.981	0.0718	0.0947	✓	303
par avance	2	13	0.68	0.969	0.0555	0.0653	✓	1265
par besoin de	7	9	1.05	0.990	0.0357	0.0630	✓	156
par ce fait	0	9	1.05	0.990	0.0320	0.0596	✓	101
par conséquent	6	6	1.30	0.998	0.0525	0.0935	✓	12234
par contre	18	12	0.72	0.989	0.0501	0.0646	×	3014
par crainte de	19	12	0.53	0.981	0.0796	0.0814	✓	534
par degrés	37	9	0.61	0.973	0.0960	0.1033	✓	1447
par dessus tout	2	10	0.73	0.991	0.0463	0.0680	✓	1433
pareil à	11	12	0.51	0.973	0.0964	0.0896	×	6787
par excellence	NO	NO	NO	NO	NO	NO	NO	1749
par faute de	4	7	0.91	0.983	0.1526	0.1476	✓	353
parfois	12	21	0.46	0.972	0.2241	0.1033	×	39445
par goût de	15	11	0.63	0.973	0.1203	0.1046	✓	143
par hasard	3	10	0.98	0.971	0.0675	0.0822	✓	7071
par instants	0	9	0.73	0.993	0.0462	0.0716	✓	1357
par manque de	0	18	0.23	0.970	0.1972	0.1047	✓	268
par mégarde	NO	NO	NO	NO	NO	NO	NO	578
parmi d'autres	25	19	0.52	0.971	0.2387	0.1121	✓	620
par moments	0	12	0.88	0.984	0.0467	0.0624	✓	2774
par ordre de	8	12	0.69	0.968	0.0725	0.0777	✓	877
par peur de	0	11	0.59	0.987	0.0564	0.0716	✓	268

LIST OF FORMS

Form	Lat.	Growth	Slope	r^2	χ^2	C.'s V	Check	# occ.
par précaution	NO	NO	NO	NO	NO	NO	NO	241
par rapport à (i)	0	10	0.97	0.978	0.0422	0.0650	✓	5290
par rapport à (ii)	2	9	0.81	0.970	0.0594	0.0812	✓	5290
par souci de	4	10	1.12	0.967	0.0789	0.0888	✓	186
par surcroît	19	12	0.59	0.982	0.0586	0.0699	✓	498
particulièrement (i)	14	18	0.51	0.982	0.0753	0.0647	✓	12784
particulièrement (ii)	3	7	1.18	0.978	0.0486	0.0833	✓	12784
par voie de	NO	NO	NO	NO	NO	NO	NO	976
par voie de conséquence	0	10	0.47	0.968	0.1316	0.1147	✓	130
petit à petit	3	10	0.77	0.985	0.0857	0.0926	✓	1547
peu à peu (i)	11	8	0.94	0.970	0.0638	0.0893	✓	16450
peu à peu (ii)	0	10	0.80	0.970	0.0736	0.0858	✓	16450
peu s'en faut	0	9	0.92	0.982	0.0498	0.0744	✓	221
pour ainsi dire	0	15	0.86	0.973	0.2276	0.1232	×	7704
pour autant	0	13	0.79	0.994	0.0116	0.0299	✓	457
pour finir	23	15	0.63	0.982	0.0759	0.0711	✓	838
pour le coup	NO	NO	NO	NO	NO	NO	NO	464
pour l'essentiel	0	9	0.97	0.985	0.0282	0.0560	✓	284
pour le moment	0	13	0.39	0.971	0.1255	0.0983	✓	2986
pour l'heure	NO	NO	NO	NO	NO	NO	NO	546
pour l'instant	11	14	0.63	0.988	0.0450	0.0567	✓	1859
pour ma part	5	6	1.12	0.997	0.1018	0.1303	✓	2744
pour peu que	0	10	0.74	0.977	0.0645	0.0803	✓	2479
pour surcroît de	NO	NO	NO	NO	NO	NO	NO	90
pourtant que (i)	0	6	1.05	0.989	0.1257	0.1447	✓	4220
pourtant que (ii)	0	6	1.70	0.989	0.0385	0.0801	✓	4220
pour tout dire	2	7	1.14	0.986	0.0781	0.1056	✓	655
pour un temps	NO	NO	NO	NO	NO	NO	NO	1333
présentement	NO	NO	NO	NO	NO	NO	NO	2683
probablement (i)	2	8	1.22	0.981	0.0568	0.0843	✓	8497
probablement (ii)	2	10	0.70	0.981	0.0700	0.0837	✓	8497
proprement	4	6	1.05	0.989	0.0825	0.1173	✓	9817
principalement	17	9	1.03	0.970	0.0497	0.0743	✓	6695
progressivement	3	9	0.59	0.978	0.1325	0.1213	×	2235
quand mme	0	18	0.43	0.972	0.1269	0.0840	✓	12171
quant à	5	15	0.37	0.966	0.1417	0.0972	×	20878

LIST OF FORMS

Form	Lat.	Growth	Slope	r^2	χ^2	C.'s V	Check	# occ.
quant à cela	NO	NO	NO	NO	NO	NO	NO	91
quant à moi	NO	NO	NO	NO	NO	NO	NO	4875
que dalle	NO	NO	NO	NO	NO	NO	NO	163
quelquefois	11	9	1.11	0.967	0.0589	0.0809	✓	34408
quelque part	NO	NO	NO	NO	NO	NO	NO	6454
relatif à	12	10	0.57	0.980	0.1375	0.1173	×	2850
relativement à	NO	NO	NO	NO	NO	NO	NO	1469
rien de plus	NO	NO	NO	NO	NO	NO	NO	1537
sans ambages	1	13	0.46	0.969	0.1474	0.1065	✓	130
sans commune mesure	2	9	1.12	0.986	0.0433	0.0694	✓	112
sans crier gare	0	13	0.55	0.972	0.0651	0.0708	✓	211
sans détour	6	15	0.51	0.970	0.1423	0.0974	✓	467
sans façon	NO	NO	NO	NO	NO	NO	NO	650
sans tenir compte de	5	8	0.85	0.983	0.0655	0.0905	✓	143
sauf	5	10	0.82	0.965	0.0660	0.0812	✓	11138
sauf si	3	12	0.74	0.994	0.0339	0.0532	✓	247
sauf que	0	14	0.41	0.968	0.1141	0.0903	✓	910
selon moi	1	13	0.39	0.982	0.1292	0.0997	✓	1055
si besoin est (i)	5	6	2.30	0.992	0.0231	0.0620	✓	106
si besoin est (ii)	3	6	2.27	0.995	0.0239	0.0631	✓	106
si bien que	4	10	0.60	0.970	0.0905	0.0951	✓	4831
si ça se trouve	0	8	0.94	0.974	0.0640	0.0894	✓	144
s'il en est	NO	NO	NO	NO	NO	NO	NO	88
si possible	22	13	0.72	0.965	0.0801	0.0785	✓	760
soit dit en passant	NO	NO	NO	NO	NO	NO	NO	276
soudain	22	20	0.47	0.970	0.1051	0.0725	×	3498
soudainement	1	9	1.08	0.968	0.0438	0.0698	✓	94
sous peu	0	6	1.83	0.993	0.0165	0.0524	✓	291
sous prétexte de	0	9	1.03	0.976	0.0486	0.0735	✓	2341
sous prétexte que	6	10	0.64	0.977	0.0928	0.0963	✓	1364
sous réserve que	NO	NO	NO	NO	NO	NO	NO	89
souventes fois	NO	NO	NO	NO	NO	NO	NO	530
spécialement	NO	NO	NO	NO	NO	NO	NO	3764
sur ce thème	NO	NO	NO	NO	NO	NO	NO	130
sur le champ	9	10	1.00	0.975	0.0834	0.0913	✓	5152
sur le moment	8	16	0.38	0.992	0.0621	0.0623	✓	715

LIST OF FORMS

Form	Lat.	Growth	Slope	r^2	χ^2	C.'s V	Check	# occ.
sur le sujet de	0	10	0.98	0.969	0.0834	0.0913	✓	292
sur le point de	13	18	0.28	0.967	0.1737	0.0982	✓	3321
sur l'heure	NO	NO	NO	NO	NO	NO	NO	720
sur l'instant	0	15	0.44	0.971	0.1206	0.0897	✓	162
un de ces jours	5	6	1.31	0.996	0.0442	0.0858	✓	983
une sorte de (i)	7	13	0.51	0.969	0.1208	0.0964	✓	31306
une sorte de (ii)	2	8	1.29	0.991	0.0396	0.0704	✓	31306
tandis que	BUG	BUG	BUG	BUG	BUG	BUG	BUG	39303
tant et plus	NO	NO	NO	NO	NO	NO	NO	155
tel quel	4	8	1.07	0.983	0.0405	0.0712	✓	985
tour à tour	11	22	0.37	0.982	0.1513	0.0829	✓	4480
tout à coup	3	9	1.11	0.983	0.0424	0.0686	✓	20468
tout à fait	20	15	0.73	0.971	0.1452	0.0984	×	25611
tout à l'heure (i)	6	12	0.57	0.967	0.0822	0.0828	✓	12853
tout à l'heure (ii)	4	13	0.71	0.977	0.0717	0.0743	✓	12853
tout au long de	12	13	0.71	0.974	0.0842	0.0805	✓	1363
tout au plus	6	23	0.33	0.971	0.1487	0.0804	✓	2954
tout bien considéré	2	6	1.16	0.994	0.0453	0.0869	✓	152
tout compte fait	NO	NO	NO	NO	NO	NO	NO	390
tout court	NO	NO	NO	NO	NO	NO	NO	1149
tout de mme (i)	8	8	0.85	0.979	0.0758	0.0973	✓	13315
tout de mme (ii)	26	13	0.74	0.991	0.0166	0.0357	✓	13315
tout du long	NO	NO	NO	NO	NO	NO	NO	302
toutefois	NO	NO	NO	NO	NO	NO	NO	20576
tout juste	17	14	0.46	0.968	0.0894	0.0799	✓	2055
tout juste de	NO	NO	NO	NO	NO	NO	NO	197
tout plein de	NO	NO	NO	NO	NO	NO	NO	1014
tout sauf	3	10	0.69	0.968	0.1026	0.1013	✓	158
tout spécialement	0	12	0.63	0.969	0.0948	0.0889	✓	164
tout un chacun	0	11	0.70	0.975	0.0812	0.0859	✓	260
très très	NO	NO	NO	NO	NO	NO	NO	356
une espèce de	16	8	1.17	0.976	0.0501	0.0791	✓	12365
un lendemain	8	8	0.56	0.993	0.1277	0.1263	✓	505
un petit peu	0	15	0.52	0.973	0.1000	0.0816	✓	692
un surcroît de	NO	NO	NO	NO	NO	NO	NO	454
un tas de	23	9	1.30	0.990	0.0243	0.0520	✓	4352

LIST OF FORMS

**Form**	**Lat.**	**Growth**	**Slope**	$\mathbf{r}^2$	χ^2	**C.'s V**	**Check**	**# occ.**
venir de	20	20	0.38	0.973	0.1164	0.0763	×	35884
vis à vis de	4	17	0.37	0.978	0.0966	0.0754	✓	3384
voilà	0	17	0.77	0.975	0.0470	0.0526	✓	90090
vu que	0	11	1.00	0.973	0.0463	0.0649	✓	1230
zut	0	10	0.99	0.987	0.0422	0.0650	✓	525

REFERENCES

[1] Sidney Redner, *A guide to first-passage processes* (Cambridge University Press, 2001)

[2] Bruno Gaume, Karine Duvignau, and Martine Vanhove, "Semantic associations and confluences in paradigmatic networks," From Polysemy to Semantic Change Towards a typology of lexical semantic associations, John Benjamins, 233–264(2008)

[3] Quentin Michard and J-P Bouchaud, "Theory of collective opinion shifts: from smooth trends to abrupt swings," The European Physical Journal B-Condensed Matter and Complex Systems **47**, 151–159 (2005)

[4] Richard A Blythe and William Croft, "S-curves and the mechanisms of propagation in language change," Language **88**, 269–304 (2012)

[5] George Lakoff and Mark Johnson, *Metaphors we live by* (University of Chicago press, 2008 [1980])

[6] Bernd Heine, *Cognitive foundations of grammar* (Oxford University Press, 1997)

[7] Sara Graça Da Silva and Jamshid J Tehrani, "Comparative phylogenetic analyses uncover the ancient roots of Indo-European folktales," Royal Society open science **3**, 150645 (2016)

[8] Uriel Weinreich, William Labov, and Marvin I Herzog, *Empirical foundations for a theory of language change* (University of Texas Press, 1968)

[9] April MS McMahon, *Understanding language change* (Cambridge University Press, 1994)

[10] Daniel Nettle, "Using social impact theory to simulate language change," Lingua **108**, 95–117 (1999)

[11] Jinyun Ke, Tao Gong, and William SY Wang, "Language change and social networks," Communications in Computational Physics **3**, 935–949 (2008)

[12] Elizabeth Closs Traugott, "On the rise of epistemic meanings in English: An example of subjectification in semantic change," Language, 31–55(1989)

[13] Steve Nicolle, "A relevance theory perspective on grammaticalization," Cognitive Linguistics **9**, 1–36 (1998)

[14] Christiane Marchello-Nizia, *Grammaticalisation et changement linguistique* (De Boeck-Duculot, 2006)

[15] ATILF, "FRANTEXT textual database, http://www.frantext.fr.." (Octobre 2014)

[16] Christiane Marchello-Nizia, "L'oral représenté: un accès construit à une face cachée des langues 'mortes'," (Peter Lang, 2012) pp. 247–264

[17] Haim Dubossarsky, Daphna Weinshall, and Eitan Grossman, "Verbs change more than nouns: a bottom-up computational approach to semantic change," Lingue e linguaggio **15**, 7–28 (2016)

[18] Eitan Adam Pechenick, Christopher M Danforth, and Peter Sheridan Dodds, "Characterizing the Google Books corpus: Strong limits to inferences of socio-cultural and linguistic evolution," PloS one **10**, e0137041 (2015)
